# Functionalisation of vitamin B_12_ derivatives with a cobalt β-phenyl ligand boosters antimetabolite activity in bacteria†

**DOI:** 10.1039/d2ra05748d

**Published:** 2022-10-07

**Authors:** Christopher Brenig, Paula Daniela Mestizo, Felix Zelder

**Affiliations:** Department of Chemistry, University of Zurich Winterthurerstrasse 190 CH 8057 Zurich Switzerland felix.zelder@chem.uzh.ch https://www.felix-zelder.net +41 44 635 6803

## Abstract

This study describes the syntheses of four singly- and two doubly-modified vitamin B_12_ derivatives for generating antimetabolites of *Lactobacillus delbrueckii* (*L. delbrueckii*). The two most potent antagonists, a Co_β_-phenyl-cobalamin-*c*,8-lactam and a 10-bromo-Co_β_-phenylcobalamin combine a *c*-lactam or 10-bromo modification at the “eastern” site of the corrin ring with an artificial organometallic phenyl group instead of a cyano ligand at the β-site of the cobalt center. These two doubly-modified B_12_ antagonists (10 nM) inhibit fully B_12_-dependent (0.1 nM) growth of *L. delbrueckii*. In contrast to potent 10-bromo-Co_β_-phenylcobalamin, single modified 10-bromo-Co_β_-cyanocobalamin lacking the artificial organometallic phenyl ligand does not show any inhibitory effect. These results suggest, that the organometallic β-phenyl ligand at the Co center ultimately steers the metabolic effect of the 10-bromo-analogue.

## Abstract 2

This study describes the syntheses of four singly- and two doubly-modified vitamin B_12_ derivatives for generating antimetabolites of *Lactobacillus delbrueckii* (*L. delbrueckii*).

## Graphical abstract

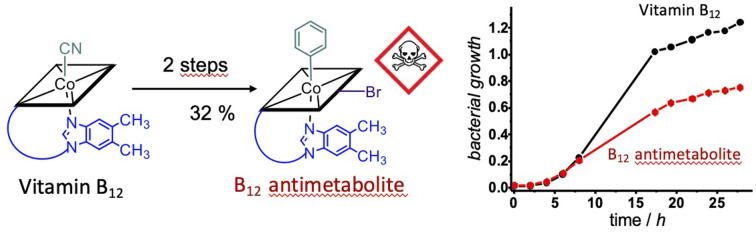

## 1. Introduction

**Fig. 1:**
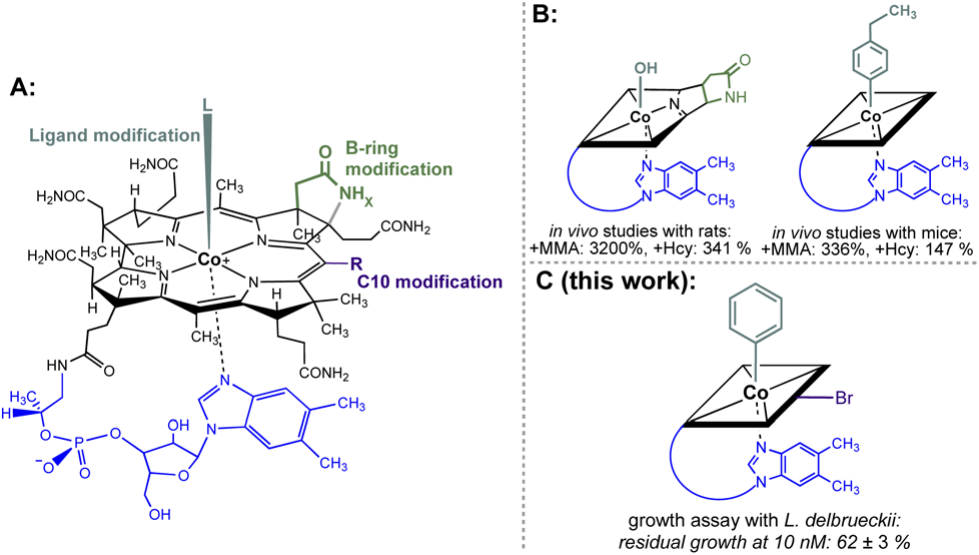
(A) General structural formula of cobalamins (Cbls; L = CN, R = H, *x* = 2 for vitamin B_12_, 1; the natural f-side chain of Cbls is depicted in blue). The upper (β-) axial ligand (turquoise), B-ring subunit (green) and the C10 position (violet) are highlighted as target sites for chemical modification. (B) Schematic depictions of B_12_ antimetabolites with a single modification at either the B-ring (left) or upper-ligand (right) modification. The effect on the increase of important biomarkers MMA (methylmalonic acid) and Hcy (homocysteine) of B_12_-dependent metabolism in mammals is indicated.^9,10^ (C) Schematic depiction of the most potent B_12_ antimetabolite of *L. delbrueckii* combining two chemical modifications at the upper ligand and the C10 position developed in this work.

Non-functional analogues of vitamins and vitamin building blocks represent an important class of drugs and drug candidates for treating different classes of diseases ranging from bacterial and fungal infections to human cancer.^1–3^ In the first half of the 20th century, prontosil was introduced as the first commercially available antimicrobial agent and saved millions of lives.^4^ The sulfonamide-based drug targets effectively bacterial, but not human biosynthesis of vitamin B_9_ (folic acid) explaining its selective therapeutic effect. Modified folic acid derivatives such as methotrexate, trimetrexate or pemetrexed represent other examples of important antibacterial and anticancer agents by inhibiting folic acid-dependent enzymatic transformations.^1,5,6^ In contrast to these folate-based drugs, modified cobalamin (vitamin B_12_) derivatives have not been developed so far to approved antibacterial or anticancer drugs.^7^ Nevertheless, some singly-modified vitamin B_12_ derivatives showed promising inhibitory effects on B_12_-dependent pathways.^8^ For example, B-ring-modified hydroxycobalamin-*c*,8-lactam (Fig. 1B left) and β-ligand-modified 4-ethylphenylcobalamin (Fig. 1B right) decreased significantly the activities of liver l-methylmalonyl coenzyme A mutase and methionine synthase as indicated by elevated plasma methylmalonic acid concentration and total homocysteine concentration in rodents.^9,10^ In contrast to these important results, bi-functionalised B_12_ derivatives combining two instead of a single modification were not explored so far in a systematic fashion.

Herein we report on the antibacterial activity of doubly-modified B_12_ derivatives. It is demonstrated in a proof-of-concept study with *L. delbrueckii*, that a specific second structural modification (*i.e.* with an artificial organometallic phenyl instead of a cyano ligand at the β-side of the Co^III^ centre) steers the metabolic effect toward the desired activity as an antimetabolite.

## 2. Results and discussion

### 2.1 Syntheses and characterisation of modified vitamin B_12_ derivatives

**Scheme 1:**
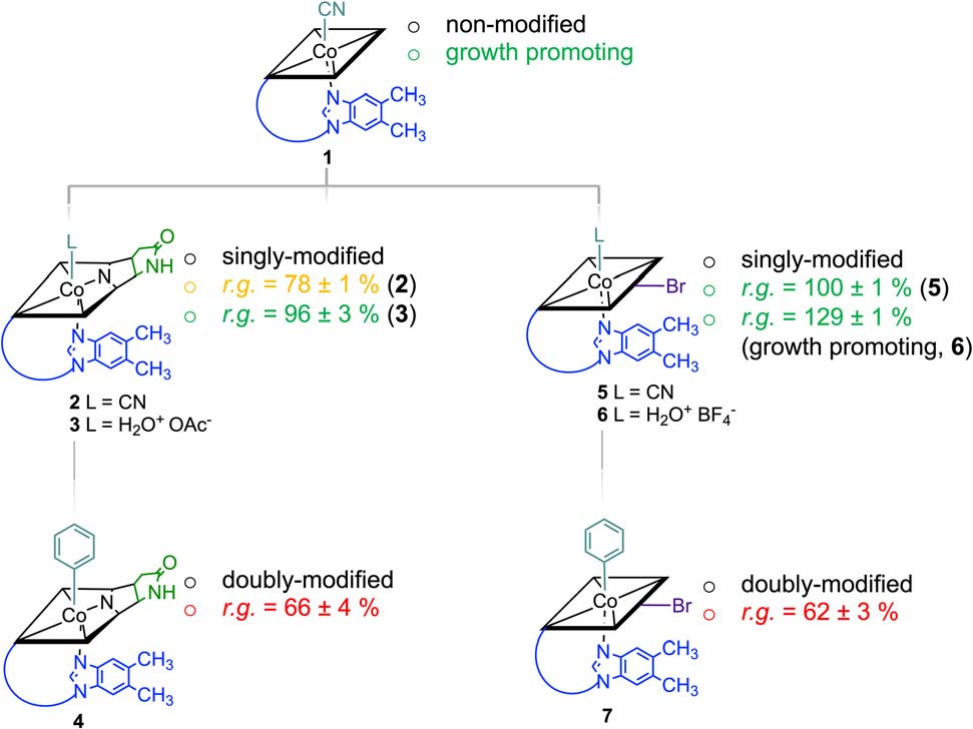
Cascade of multiple chemical modifications of Cbls and their physiologic effect (promotion of growth or activity as antimetabolite; r.g. = residual bacterial growth compared to a B_12_-only control group) on a *L. delbrueckii* bacterial culture (the strength of the effect is indicated with colors; green: growth promoting; orange: medium inhibition of growth; red: strong inhibition of growth). The scheme does not indicate the course of chemical reactions. Charges omitted for clarity.

Starting from vitamin B_12_ (1; Fig. 1A) we prepared two novel bi-functionalized Cbls (4 and 7; Scheme 1) using established synthetic protocols.^11^ In particular, we combined in the analogues either a *c*,8-lactam^11^ (Scheme 1 left) or C10–Br^12^ (Scheme 1 right) modification at the corrin ring with a β-phenyl ligand at the Co-center (Scheme 1, bottom line). These combinations were purposefully chosen because the selected transformations (i) induced biological effects in previous studies when incorporated into the Cbl scaffold (Fig. 1B) and (ii) provide analogs in high purity under mild conditions.^9,10^

**Scheme 2:**
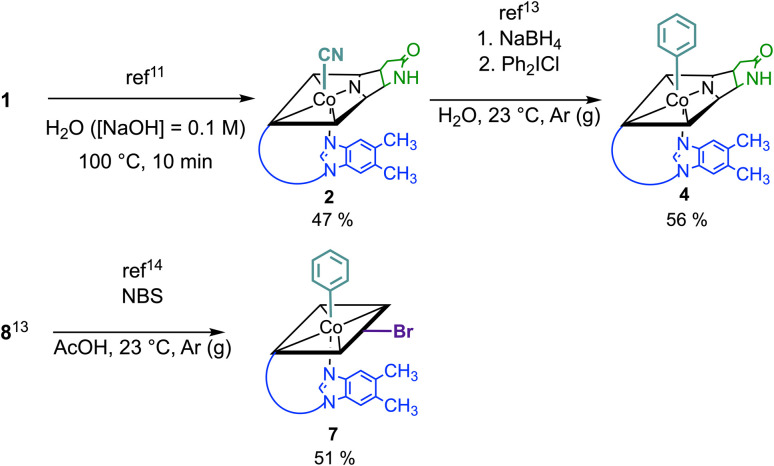
Top: synthesis of Co_β_-phenyl-Cbl-*c*,8-lactam (4) from vitamin B_12_ (1) in a two-step procedure *via* Co_β_-cyano-Cbl-*c*,8-lactam (2) applying selective and high-yielding chemical transformations in aqueous media. Bottom: synthesis of 10-bromo-Co_β_-phenylcobalamin (7) starting from Co_β_-phenylcobalamin (8).

Syntheses, isolation, and characterization of the two novel doubly-modified derivatives 4 and 7 and four singly-modified Cbls (2, 3, 5 and 6) are described in more detail below and in the Experimental section. In this section, we outline only the preparation and characterization of unprecedented compounds Co_β_-phenyl-Cbl-*c*,8-lactam (4; Scheme 2 top) and 10-bromo-Co_β_-phenylcobalamin (7; Scheme 2 bottom).

Vitamin B_12_ analogue 4 was synthesized in two steps starting from commercially available B_12_ in a total isolated yield of 27%. First, *c*,8-lactam 2 was prepared under basic conditions at 100 °C following a procedure of Todd *et al.*^11^ After reduction of its Co^III^ center with NaBH_4_ (10 equiv.) and subsequent treatment with diphenyl iodonium chloride (2 equiv.) in H_2_O,^13^ the organometallic target 4 was obtained. The occurrence of a pseudo molecular ion at *m*/*z* = 1404.56 ([M + H]^+^, *m*/*z*_calc_: 1404.60 for C_68_H_92_CoN_13_O_14_P^+^) in the ESI-MS spectrum of 4 indicated successful arylation at the Co center of 2, supported by the observation of a hypsochromically shifted γ-band (Δ*λ* = 18 nm) with diminished intensity (Δlog *ε* = 0.22) in the UV/vis spectrum. This spectral behavior is typical for organometallic Cbls featuring a Co^III^–C bond (Fig. S13†).^13^ For the synthesis of 10-bromo-Co_β_-phenylcobalamin (7; Scheme 2 bottom), we considered that Cbls bearing good leaving groups are prone towards reducing agents. Therefore, 10-bromo-Co_β_-cyanocobalamin (5) is not compatible with arylation conditions using NaBH_4_.^14^ Having this in mind, we brominated the previously described Co_β_-phenylcobalamin (8)^13^ with *N*-bromosuccinimide (NBS) at its C10 position according to a method of Wagner.^14^ ESI-MS analysis confirmed successful formation of 7 by the presence of its adduct ion peak at *m*/*z* = 1485.53 ([M + H]^+^) and the characteristic bromine isotopic pattern. Bromination at position C10 of 7 was further proven by the absence of the signal of the proton at C10 in the ^1^H-NMR spectrum and the absorption spectrum of 7 exhibited a characteristic redshift of the αβ-band to 537 nm.^12^

### 2.2 Bacterial growth assays

The inhibitory potential of the small library of four singly-, and two doubly modified B_12_ derivatives (2–7) was assessed with bacterial growth assays using *L. delbrueckii*.

**Fig. 2:**
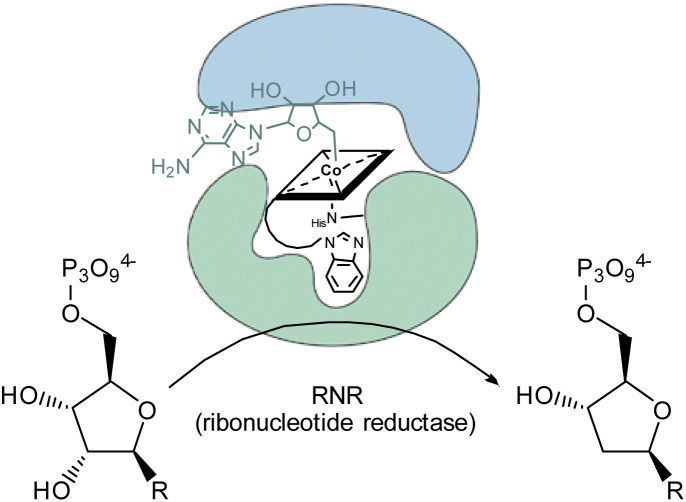
Schematic representation of the conversion of ribonucleotides to deoxyribonucleotides catalyzed by 5′-adenosylcobalamin-dependent ribonucleotide reductase in the metabolism of *L. delbrueckii*.^15^

**Fig. 3:**
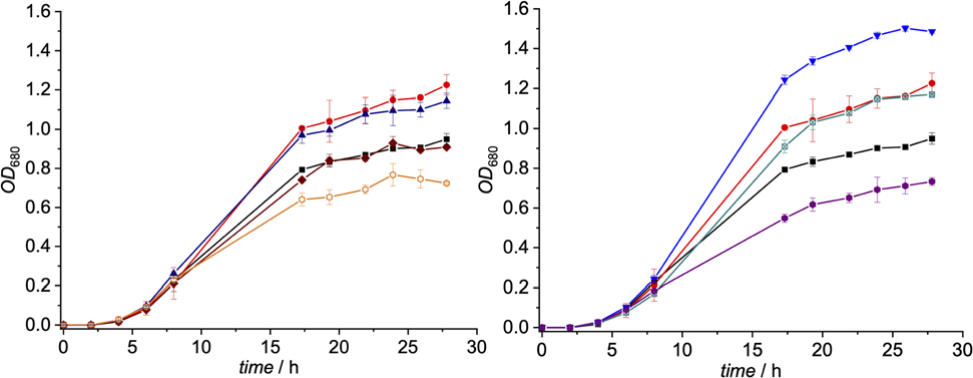
Left: growth of a suspension culture of *L. delbrueckii* in presence of derivatives 2 (brown rhombs), 3 (blue triangles) and 4 (orange hollow hexagons) (*c* = 10 nM) and B_12_ (1, *c* = 0.1 nM) *vs.* two control groups (positive control: red dots; containing only 1 (0.1 nM), negative control: black squares; containing only medium) at 37 °C (*n* = 3). Right: growth of a suspension culture of *L. delbrueckii* in presence of derivatives 5 (turquoise marked squares), 6 (inverse blue triangles) and 7 (violet hexagons) (*c* = 10 nM) and B_12_ (1, *c* = 0.1 nM) *vs.* two control groups (positive control: red dots; containing only 1 (0.1 nM), negative control: black squares; containing only medium) at 37 °C (*n* = 3).

These Gram-positive bacteria are ideal for such proof-of-concept studies since they possess ribonucleotide reductase (RNR) as the only Cbl-dependent enzyme (see Fig. 2 for details).^15–17^ Small concentrations of B_12_ (0.1 nM) in the medium are sufficient to support growth of *L. delbrueckii* (positive control; Fig. 3).

Notably, when exogenous B_12_ was absent in the assay medium (negative control), substantial residual growth (r.g.; 80 ± 1%) was still observed suggesting contamination of the B_12_-free medium with little amounts of Cbl.^17–19^ In competition assays with a 100-fold excess of analogs 2–7 (10 nM) over B_12_, *c*,8-lactam modified CNCbl 2 showed 22% inhibition (r.g. = 78 ± 1%; Fig. 3 left) of B_12_-triggered growth. Bi-functionalization of analog 2 with an additional organometallic β-phenyl ligand (*i.e.*, analog 4) strengthened further the antimicrobial activity (r.g. = 66 ± 4%; Fig. 3 left). Inhibition in this competition assay was stronger than residual growth in the absence of exogeneous B_12_ (*i.e.*, negative control) suggesting that total B_12_ in the medium was effectively outcompeted by the presence of analogue 4 (Fig. 3 left). In contrast to single modified *c*,8-lactam 2, single-modified 10-BrCNCbl (5) had no evident effects on B_12_-dependent growth (r.g. = 100 ± 1%; Fig. 3 right). This biological lethargy changed drastically upon further modification with a β-phenyl group at the Co^III^ center. Bi-functionalized 7 with a 10-Br modification was a comparably strong inhibitor (r.g. = 62 ± 3%) as bi-functionalized 4 with a *c*,8-lactam group (Fig. 3). The importance of the β-phenyl functionality at the Co^III^ center of inhibitors 4 and 7 for triggering the antimetabolic effect was further supported by studying the corresponding Co_β_-aqua derivatives 3 and 6. These analogues lacking the organometallic phenyl ligand had either negligible (r.g. (3) = 96 ± 3%, Fig. 3-left) or even growth-promoting effects (r.g. (6) = 129 ± 1%, Fig. 3 right).

The latter result suggests, that aqua derivative 7 is still recognized, internalized, and metabolized by the microorganism to a growth promoting, enzymatically active organometallic AdoCbl cofactor (Fig. 3). In contrast, this reductive biological transformation is apparently not possible for 2, 4 and 7 containing σ-donating cyano or phenyl ligands in addition to a strucurally altered Cbl scaffold.^20^ Although we propose herein inhibition of RNR with inhibitors 2, 4 and 7 (Fig. 3), the actual biological target(s) and modes of action of these B_12_ analogues have still to be unraveled in future biological studies. So far, our proof-of-concept studies clearly demonstrate that the second, ligand-centered modification of the analogues with a β-phenyl ligand significantly steers antimetabolite activity. Replacement of this ligand with a weakly coordinating aqua ligand effected complete reversal of the antimetabolite activity of both bi-functionalized derivatives.

## 3. Conclusions

We have synthesized and tested antibacterial activity of four single- and two novel bi-functionalised B_12_ analogues. B_12_-dependent growth studies with *L. delbrueckii* showed strikingly that doubly modified Co_β_-phenyl-cobalamin-*c*,8-lactam and 10-bromo-Co_β_-phenylcobalamin were the most potent antagonists. Of note, inhibition was even stronger than residual growth in the absence of exogenous B_12_. Moreover, these studies demonstrated strikingly, that the second, β-axial modification significantly steers the metabolic effect. In particular, the incorporation of an organometallic β-phenyl ligand at the cobalt center either empowered the inhibitory potential of CNCbl-*c*,8-lactam or more interestingly, induced antimetabolic activity in an erstwhile innocent 10-brominated Cbl analogue.

## 4. Experimental section

### 4.1 General

Chemicals were of reagent grade quality or better and obtained from Sigma-Aldrich, ACROS Organics, Merck or Fluka and used without further purification unless otherwise indicated. Vitamin B_12_ was obtained from Sigma-Aldrich or received as a generous gift from DSM Nutritional Products AG (Basel/Switzerland) and Prof. Em. Bernhard Jaun (ETH Zurich, Switzerland). All solvents were of reagent, analytical, HPLC or LC-MS grade, respectively, and obtained from commercial suppliers. Bi-distilled H_2_O was used in all reactions. H_2_O from a Milli-Q (Merck-Millipore) water purification system was used for UV/vis spectroscopy, mass spectrometry and when indicated. Reactions were carried out under N_2_ (g) or Ar (g) in oven-dried (100 °C) glass equipment and monitored for completion by analysing a small sample (after suitable workup) by LC-MS. Evaporation of the solvents *in vacuo* was done with the rotary evaporator (Büchi) at the given bath temperature and pressure. SepPak® RP-18 cartridges (Waters) were applied for solid phase extraction. The compounds were dissolved in H_2_O, transferred to the adsorbent, washed with H_2_O or the indicated aq. soln, followed by H_2_O, and eluted with CH_3_OH.

### 4.2 Chromatography

#### Preparative HPLC

Separations were conducted on a LaPrep Sigma HPLC system (Knauer/VWR) equipped with a UV detector, a sample collector and a Nucleosil 100-7 C18 250/40 column (Macherey-Nagel). Method A: a gradient (0 min 5.0% A, 0–30 min 80% A, 30.1–40 min 95% A, 40.1–50 min 95% A) of CH_3_CN (solvent A) *vs.* an aq. soln of 0.1% CF_3_COOH (solvent B) was applied using a flow rate of 30 mL min^−1^. Method B: a gradient (0 min 15% A, 0–30 min 80% A, 30.1–40 min 95% A, 40.1–50 min 95% A) of CH_3_OH (solvent A) *vs.* an aq. soln of K_2_HPO_4_/KH_2_PO_4_ (10 mm, pH 7.0; solvent B) was applied using a flow rate of 30 mL min^−1^. Method C: a gradient (0 min 5.0% A, 0–30 min 65% A, 30.1–40 min 95% A, 40.1–50 min 95% A) of CH_3_OH (solvent A) *vs.* an aq. soln of K_2_HPO_4_/KH_2_PO_4_ (10 mm, pH 7.0; solvent B) was applied using a flow rate of 30 mL min^−1^. LC-MS was performed on an ACQUITY UPLC system (Waters) equipped with a PDA detector and an autosampler using an ACQUITY UPLC BEH C18 Gravity 1.7 μm (2.1 mm × 50 mm) reversed phase column (Waters). The UPLC system was connected to a HCT ESI-MS spectrometer (Bruker Daltonics), operated in positive or negative mode; nebulizer pressure 60 psi, dry gas flow rate 10 L min^−1^, dry gas temperature 365 °C, scan range *m*/*z* 200–2000. Samples were dissolved in H_2_O, CH_3_OH or CH_3_CN and a total volume of 2.0 μL of the sample was analyzed; Method 1: a gradient (0 min 5.0% A, 0.5–2.0 min 5–30% A, 2.01–4.0 min 30–100% A, 4.01–5.0 min 100% A) of CH_3_CN (solvent A) *vs.* an aq. soln of 0.1% HCOOH (solvent B) was applied using a flow rate of 0.3 mL min^−1^. Method 2: a gradient (0 min 5.0% A, 0.5–2.0 min 5–30% A, 2.01–4.01 min 30–100% A, 4.01–5.0 min 100% A) of CH_3_CN (solvent A) *vs.* an aq. soln of 0.1% HCOOH (solvent B) was applied using a flow rate of 0.5 mL min^−1^. High resolution electrospray ionization mass spectrometry (HR-ESI-MS) was performed on a Dionex Ultimate 3000 UHPLC system (Thermo Fischer Scientifics, Germering, Germany) connected to a QExactive MS with a heated ESI source (Thermo Fisher Scientific, Bremen, Germany); onflow injection of 1 μL sample (*c* = approx. 50 μg mL^−1^ in the indicated solvent) with an XRS auto-sampler (CTC, Zwingen, Switzerland); flow rate 120 μL min^−1^; ESI: spray voltage 3.0 kV, capillary temperature 280 °C, sheath gas 30 L min^−1^, aux gas 8 L min^−1^, s-lens RF level 55.0, aux gas temperature 250 °C (N_2_); full scan MS in the alternating (+)/(−)-ESI mode; mass ranges 80–1200 *m*/*z*, 133–2000 *m*/*z*, or 200–3000 *m*/*z* at 70 000 resolution (full width half-maximum); automatic gain control (AGC) target of 3.00 × 10^6^; maximum allowed ion transfer time (IT) 30 ms; mass calibration to <2 ppm accuracy with Pierce® ESI calibration solns (Thermo Fisher Scientific, Rockford, USA); lock masses: ubiquitous erucamide (*m*/*z* 338.34174, (+)-ESI) and palmitic acid (*m*/*z* 255.23295, (−)-ESI).

### 4.3 Spectroscopy

#### UV/vis spectra

Cary 50 Scan spectrophotometer (Varian) or Specord 250 Plus (Analytik Jena) using 1 cm quartz cuvettes (Hellma Analytics); *λ*_max_ (log *ε*) in nm. Both ^1^H- and ^13^C-NMR spectra were carried out at 298 K in D_2_O or CD_3_OD and at 500 MHz or 126 MHz, respectively. The ^1^H-NMR spectra were performed in an AVANCE NEO 500 MHz spectrometer (Bruker) using a 5 mm-*z*-gradient RT-BBI probehead; *δ* in ppm relative to *H*DO (*δ* 4.79; corresponds to TMS (*δ* 0.00)) or C*H*D_2_OD (*δ* 3.31; corresponds to TMS (*δ* 0.00)), *J* in Hz. Spectra in D_2_O were presaturated. The ^13^C-NMR spectra were performed in an AVANCE NEO 500 MHz spectrometer (Bruker) using a 5 mm z-gradient CP-BBO probehead; *δ* in ppm relative to *C*D_3_OD (*δ* 49.0; corresponds to TMS (*δ* 0.0)), *J* in Hz.

### 4.4 Bacterial growth assays

Culture medium was prepared by dissolving MRS broth (Difco) for *Lactobacilli* (5.5 g) in Milli-Q H_2_O (100 mL) and subsequently filtered through a sterile 2.0 μm filter. The culture medium (14 mL) was inoculated with *Lactobacillus delbrueckii* subsp. *Lactis*, DSM 20355 from a micro-ring culture (previously stored at −70 °C). The closed tubes were incubated at 30 °C for 24 h. Afterwards a small sample (0.5 mL) was taken out and OD_680 nm_ was determined and typically yielded values around 1.0 after 24 h. A second culture was inoculated by addition of the inoculate (200 μL) to fresh MRS broth (14 mL), followed by incubation at 30 °C for 24 h, resulting in OD_680 nm_ = 1.5 prior to the conduction of the assay. The bacterial culture was centrifuged (5000 rpm/5 min), and the remaining pellet was suspended in H_2_O ([NaCl] = 0.9%, 14.0 mL) and incubated at 37 °C for 30 min. The resulting suspension was centrifuged again (5000 rpm/5 min), followed by two washing steps in H_2_O ([NaCl] = 0.9%, 14.0 mL) to remove remaining traces of the growth medium. Afterwards, H_2_O ([NaCl] = 0.9%, 5.0 mL) was added to the pellet and the bacterial suspension was stored at 37 °C. Vitamin B_12_ assay medium (Sigma-Aldrich, 41.5 g) was dissolved in Milli-Q H_2_O (500 mL) and the mixt. was heated to 40 °C under stirring until everything was dissolved, before Tween® 80 (1.0 mL) was added and everything was thoroughly homogenized. The pH was adjusted to 6.0 by addition of H_2_O ([NaOH] = 0.5 M) and the medium was filtered through a sterile 2.0 μm filter. Subsequently, sterilized bacterial assay glass tubes (Fisher Scientific, total volume: 7.0 mL) were filled with of B_12_ assay medium (6.5 mL), a sterile stock solution of vitamin B_12_ in Milli-Q H_2_O (6.5 μL, 1.0 nM), except for the negative control, and a sterile soln of the respective test compound in H_2_O (6.5 μL, 100 nM to 0.1 μM), except for the positive control. All tubes were finally inoculated with 40 μL of the bacterial suspension, tightly closed, and incubated at 37 °C for 28–60 h. OD_680 nm_ was monitored photometrically every 2–8 h (after through mixing of the tubes), until saturation of growth was detected. All assays were performed in triplicates and average values of OD_680 nm_ (±2*σ*) were obtained and plotted *vs.* time (*t*) in h (hours) to obtain growth curves. Residual growth values (r.g.) were estimated after 26 h and are given in% relative to the growth of the positive control group.

### 4.5 Experimental procedures

#### Co_β_-cyanocobalamin-*c*,8-lactam (2)

B-Ring lactam formation in Co_β_-cyanocobalamin (1) was performed according to lit.^11^ Briefly, 1 (100 mg, 73.8 μmol, 1.0 equiv.) was added to a soln of NaOH (1.0 M in H_2_O, 20 mL) and the soln was heated to 100 °C for 10 min. The reaction mixture was adjusted to pH 8.0 by addition of NaHCO_3_ and the crude product was extracted using SPE. Purification *via* preparative HPLC (method B) and subsequent lyophilization afforded 2 (46.6 mg, 34.4 μmol, 47%) as a red powdery solid.

UV-vis (H_2_O, *c* = 4.1 × 10^−5^ M): 279 (3.92), 308 (3.69), 321 (3.63), 360 (4.20), 518 (3.64), 549 (3.66). UPLC: *t*_ret_ = 1.70 min (method 2). ESI-MS (H_2_O/MeCN): *m*/*z* = 677.58 (100, [M + 2H]^2+^), 1353.55 (10, [M + H]^+^, *m*/*z*_calc_: 1353.56 for C_63_H_87_CoN_14_O_14_P^+^).^1^H-NMR (D_2_O, *c* = 4.1 × 10^−5^ M): 7.27 (s, *H*C7N), 7.09 (s, *H*C2N), 6.45 (s, *H*C4N), 6.31 (d, *J* = 3.0, *H*C1R), 5.93 (s, *H*C10), 4.67 (d, *J* = 3.8, ribose-C*H*OH), 4.30–4.20 (m, 2 corrin-C*H*), 4.11 (d, *J* = 8.3, corrin-C*H*), 4.07–3.99 (m, 2 corrin-C*H*), 3.91–3.85 (d-like m, H_a_ of H_2_C5R), 3.70 (dd, *J* = 12.8, 3.8, H_b_ of H_2_C5R), 3.55 (d, *J* = 14.3, H_a_ of H_2_C175), 3.36–3.27 (m, *H*C13), 2.95–2.85 (m, H_b_ of H_2_C175, corrin-C*H*), 2.79–2.41 (m, 5 corrin-C*H*_2_) superimposed by 2.54 (s, *H*_3_C151) and 2.52 (s, *H*_3_C51), 3.40–2.31 (m, 5 corrin-C*H*_2_), 2.10–1.73 (m, 4 corrin-C*H*_2_) superimposed by 2.24 (s, dmbi-C*H*_3_), 2.22 (s, dmbi-C*H*_3_) and 1.84 (s, *H*_3_C7A), 1.42 (s, *H*_3_C12A), 1.37 (s, *H*_3_C2A), 1.34–1.23 (m, corrin-C*H*_2_) superimposed by 1.32 (s, *H*_3_C17B), 1.21 (d, *J* = 6.0, *H*_3_C177), 1.12 (s, *H*_3_C12B), 0.45 (s, *H*_3_C1A). Assignments were made in comparison with data from lit.^21,22^

#### Co_β_-aquacobalamin-*c*,8-lactam acetate (3)

A soln of 2 (5.0 mg, 3.7 μmol) in H_2_O (1.0 mL) was degassed by purging with N_2_ (g) for 15 min, before a soln of NaBH_4_ (1.5 mg, 40 μmol, 11 equiv.) in H_2_O (0.1 mL) was added. The resulting mixt. was stirred at 23 °C for 10 min, until a color change to dark violet occurred. Subsequently, AgOAc (1.2 mg, 7.2 μmol, 1.9 equiv.) was added, resulting in formation of a white precipitate. The precipitate was filtered off and a gentle stream of air was passed through the remaining soln, upon which it turned red. LC-MS analysis (method 2) revealed formation of the aquo complex 3 as the sole product. The product was extracted using SPE, washed with an aq. soln of NH_4_OAc (0.1 M, 10 mL) and eluted with CH_3_OH. The solvent was evaporated *in vacuo* (200 mbar, 40 °C) and the residue was re-dissolved in H_2_O (1.5 mL) and lyophilized overnight to afford 3 (5.1 mg, 3.7 μmol, quant.).

UV/vis (H_2_O, *c* = 2.2 × 10^−5^ M): 276 (sh., 4.38), 290 (sh., 4.29), 349 (4.39), 404 (sh., 3.76), 495 (3.96), 523 (3.94). UPLC: *t*_ret_ = 1.30–1.60 min (method 2). ESI-MS (H_2_O/MeCN): *m*/*z* = 664.02 (100, [M–H_2_O + H]^2+^, *m*/*z*_calc_: 663.78 for C_62_H_87_CoN_13_O_14_P^2+^).^1^H-NMR (CD_3_OD, *c* = 7.3 × 10^−3^ M): 7.87 (d, *J* = 3.7, O*H*), 7.22 (s, C*H*7N), 6.96 (s, *H*C2N), 6.59 (s, *H*C4N), 6.21 (d, *J* = 3.1, *H*C1R), 6.19 (s, *H*C10), 4.71–4.63 (m, ribose-C*H*OH), 4.36 (d, *J* = 8.2 Hz, corrin-C*H*), 4.19–4.15 (m, corrin-C*H*, ribose-C*H*), 4.13–4.09 (m, corrin-C*H*), 3.95 (dd, *J* = 12.7, 2.9, H_a_ of H_2_C5R), 3.85 (d, *J* = 9.5, H_a_ of corrin-CH_2_), 3.78 (dd, *J* = 12.7, 4.1, H_b_ of H_2_C5R), 3.75–3.69 (m, corrin-C*H*), 3.51 (d, *J* = 10.7, H_b_ of corrin-CH_2_), 3.36 (s, superimposed by CHD_2_OD signal), 3.11–3.07 (m, corrin-C*H*), 3.04 (d, *J* = 17.7, corrin-C*H*), 2.88 (d, *J* = 17.7, corrin-C*H*), 2.85–2.73 (m, corrin-C*H*_2_), 2.72 (s, corrin-C*H*_3_), 2.69–2.48 (m, 5 corrin-C*H*_2_) superimposed by 2.63 (2s, corrin-C*H*_3_, C*H*_3_COO), 2.45–2.37 (m, H_a/b_ of corrin-CH_2_), 2.33 (s, dmbi-C*H*_3_), 2.29 (s, dmbi-C*H*_3_), 2.23–2.10 (m, 3 corrin-C*H*_2_), 1.97 (s, corrin-C*H*_3_), 1.94–1.82 (m, corrin-C*H*_2_, *H*_a/b_ of corrin-CH_2_), 1.66 (d, *J* = 7.6, H_a/b_ of corrin-CH_2_), 1.58 (s, corrin-C*H*_3_), 1.53 (s, corrin-C*H*_3_), 1.51–1.41 (m, corrin-C*H*_2_), 1.38 (s, corrin-C*H*_3_), 1.34 (s, corrin-C*H*_3_), 1.29 (d, *J* = 6.4, *H*_3_C177), 0.53 (s, *H*_3_C1A). Assignments were made in comparison with data from lit.^22^

#### Co_β_-phenylcobalamin-*c*,8-lactam (4)

In a Schlenk tube, 2 (25.0 mg, 18.5 μmol) was dissolved in H_2_O (2.0 mL) and the solution was degassed using pump–freeze–thaw cycling (three cycles). To the degassed solution, NaBH_4_ (6.90 mg, 182 μmol, 9.8 equiv.) was added under N_2_ counterflow. The mixture was stirred at 23 °C for 30 min, until the solution turned dark brown. Subsequently, diphenyliodonium chloride (11.4 mg, 36.0 μmol, 1.9 equiv.) were added and the mixture was stirred at 23 °C for further 60 min, until LC-MS analysis (method 1) showed full conversion of the starting material and formation of two products with *m*/*z* = 1404.6 in a *ca.* 3 : 1 ratio, corresponding to the isomeric forms of 4. The raw products were isolated using SPE. Subsequent purification *via* prep. HPLC (method A), followed by crystallization from aqueous acetone, delivered Co_β_-phenylcobalamin-*c*,8-lactam (4, 14.5 mg, 10.3 μmol, 56%) as pale red crystals and, after precipitation from MeOH/ethyl acetate, its side product 4a (Scheme S3, ESI†) (1.85 mg, 1.30 μmol, 7%) as an orange powder. 4a (Scheme S3, ESI†) was tentatively assigned according to ref. 13 and not further characterized.

UV-vis (H_2_O, *c* = 3.9 × 10^−5^ M): 283 (4.09), 342 (3.98), 370 (3.80), 470 (sh., 3.55), 517 (3.73). UPLC: *t*_ret_ = 2.45 min (method 1). HRMS (ESI+): *m*/*z* = 702.79905 (100, [C_68_H_91_O_14_N_13_CoP + 2H]^2+^, *m*/*z*_calc_: 702.80150), *m*/*z* = 1404.59518 (50, [M + H]^+^, *m*/*z*_calc_ = 1404.59564). ^1^H-NMR (D_2_O, *c* = 1.2 × 10^−2^ M) *δ* 7.34 (s, *H*C2N), 7.26 (s, *H*C7N), 6.81 (d, *J* = 5.7 Hz, *H*C4L), 6.76 (t, *J* = 6.9 Hz, *H*C3L, *H*C5L), 6.66 (s, *H*C4N), 6.29 (d, *J* = 3.1 Hz, *H*CR1), 5.89 (s, *H*C10), 5.79 (d, *J* = 7.8 Hz, *H*C2L, *H*C6L), 4.70 (td, *J* = 8.4, 4.3 Hz, *H*C3R), 4.42–4.33 (m, *H*C176), 4.30 (t, *J* = 3.7 Hz, *H*C2R), 4.20 (d, *J* = 8.4 Hz, *H*C3), 4.18–4.13 (m, *H*C4R), 3.97 (dd, *J* = 12.9, 2.5 Hz, H_a_ of H_2_C5R), 3.78 (dd, *J* = 13.0, 4.2 Hz, H_b_ of H_2_C5R), 3.57 (dt, *J* = 14.0, 2.3 Hz, H_a_ of H_2_C175), 3.43 (d, *J* = 10.3 Hz, *H*C13), 3.36 (d, 10.0 Hz, *H*C19), 3.05–2.90 (m, H_a_ and H_b_ of H_2_C71, H_b_ of H_2_C175), 2.79 (s, *H*_3_C51), 2.76–2.74 (t-like, *J* = 5.9 Hz, *H*C18), 2.70 (s, *H*_3_C151), 2.68–2.61 (m, H_a_ and H_b_ of H_2_C132, H_a_ of H_2_C171), 2.58–2.47 (m, *H*_2_C32, H_a_ of H_2_C172), 2.44 (d, *J* = 5.9 Hz, *H*_2_C181), 2.34 (s, *H*_3_C10N), 2.31–2.23 (t-like, *H*_3_C11N, H_b_ of H_2_172), 2.19–1.99 (m, *H*_2_C82, H_a_ of H_2_C131, *H*_2_C31), 1.97–1.73 (m, H_b_ of H_2_C131, *H*_2_C21, H_b_ of H_2_C171) superimposed by 1.92 (s, *H*_3_C7A), 1.60–1.46 (m, H_a_ of H_2_C81), 1.42 (s, *H*_3_C12A), 1.29 (s, *H*_3_C2A), 1.24 (d, *J* = 6.3 Hz, *H*_3_C177), 1.18 (s, *H*_3_C17B), 1.16–1.09 (m, H_b_ of H_2_C81), 0.93 (s, *H*_3_C12B), 0.55 (s, *H*_3_C1A). ^13^C-NMR (D_2_O, *c* = 1.2 × 10^−2^ M) *δ* 178.2 (C133), 178.0 (C33), 177.6 (C11), 176.5 (C72), 176.3 (C16), 176.3 (C83), 176.1 (C22), 176.0 (C182), 175.7 (C4), 174.9 (C173), 168.1 (C9), 163.8 (C14), 162.3 (C6), 144.3 (C1L), 142.4 (C2N), 137.7 (C8N), 133.9 (C5N), 133.0 (C2L, C6L), 131.8 (C6N), 130.1 (C9N), 127.3 (C3L, C5L), 124.8 (C4L), 118.8 (C4N), 110.9 (C7N), 106.5 (C5), 105.9 (C15), 90.4 (C10), 86.8 (C1R), 85.6 (C1), 81.7 (C4R), 75.6 (C19), 74.8 (C8), 72.9 (C3R), 72.5 (C176), 68.7 (C2R), 60.3 (C5R), 59.0 (C17), 56.0 (C3), 53.2 (C13), 50.9 (C7), 47.6 (C12), 46.1 (C2), 45.2 (C175), 43.7 (C71), 41.5 (C21), 38.4 (C18), 35.2 (C32), 34.4 (C132), 32.8 (C171), 32.2 (C172), 31.7 (C181), 29.7 (C12B), 29.4 (C81), 29.0 (C82), 27.9 (C131), 25.2 (C31), 21.0 (C1A), 20.0 (C7A), 19.9 (C12A), 19.8 (C10N), 19.1 (C11N), 18.8 (C177), 17.1 (C17B), 16.8 (C51), 16.2 (C2A), 15.2 (C151). Assignments were made based on 2D NMR studies (DQF-COSY, HSQC, HMBC, NOESY) and comparison with data from lit.^13^

#### 10-Bromo-Co_β_-cyanocobalamin (5)

C10-bromination of 1 was performed based on a procedure published earlier by our group.^12^ Under vigorous stirring, 1 (100 mg, 74 μmol, 1.0 equiv.) was dissolved in glacial acetic acid (3.0 mL). After purging with nitrogen for 10 min, NBS (13.0 mg, 74 μmol, 1.0 equiv.) was added to the stirred soln in small portions over time (3 h) at 23 °C. The solution turned dark purple upon addition. After complete addition, LC-MS analysis (method 1) showed full conversion of the starting material. The reaction mixture was diluted with H_2_O (25 mL) and corrinoid material was extracted with SPE. Solvent was subsequently removed under reduced pressure and the crude product was purified by preparative HPLC (method C) affording 5 (72.2 mg, 50 μmol, 68%) as a dark purple powder after lyophilization.

UV-vis (H_2_O, *c* = 4.9 × 10^−5^ M): 283 (3.69), 290 (3.71), 367 (4.13), 416 (sh., 3.15), 553 (3.51), 577 (3.55). UPLC: *t*_ret_ = 2.05 min (method 1). ESI-MS (H_2_O/MeOH): *m*/*z* = 718.21 (100, [M + 2H]^2+^), 1435.45 (11, [M + H]^+^, *m*/*z*_calc_: 1435.48 for C_63_H_88_BrCoN_14_O_14_P^+^). ^1^H-NMR (D_2_O, *c* = 4.2 × 10^−3^ M): 7.27 (s, *H*C7N), 7.09 (s, *H*C2N), 6.48 (s, *H*C4N), 6.34 (d, *J* = 3.0, *H*C1R), 4.74–4.70 (m, ribose-C*H*OH), 3.37 (dd, *J* = 8.3, 6.8, ribose-C*H*OH), 4.31–4.24 (m, corrin C*H*, ribose-C*H*OH), 4.20 (d, *J* = 9.0, corrin-C*H*), 4.03 (t, *J* = 9.0, 2H), 3.93–3.87 (m, 1H), 3.73 (dd, *J* = 14.2, 3.4), 3.59 (d, *J* = 14.3 Hz, corrin-C*H*), 3.35 (d, *J* = 9.0, corrin-C*H*), 2.94 (dd, *J* = 14.3, 9.8, corrin-C*H*), 2.76–2.59 (m, 5 corrin-C*H*_2_), 2.58–2.52 (m, 2 corrin-C*H*_2_) superimposed by 2.57 (s, corrin-C*H*_3_) and 2.54 (s, corrin-C*H*_3_), 2.40–2.15 (m, 2 corrin-C*H*_2_), superimposed by 2.25 (s, corrin-C*H*_3_) and 2.23 (s, corrin-C*H*_3_), 2.11–1.75 (m, 3 corrin-C*H*_2_) superimposed by 1.89 (s, corrin-C*H*_3_) and 1.79 (s, corrin-C*H*_3_), 1.36 (s, corrin-C*H*_3_), 1.35 (s, corrin-C*H*_3_), 1.29 (s, corrin-C*H*_3_), 1.23 (d, *J* = 6.0, *H*_3_C177), 1.21–1.06 (m, corrin-C*H*_2_), 0.35 (s, corrin-C*H*_3_). Data is in agreement with lit.^12^

#### 10-Bromo-Co_β_-aquacobalamin tetrafluoroborate (6)

was synthesized *via* intermediate 10-bromo-Co_β_-phenylethynylcobalamin (10-BrPhEtyCbl), as reported earlier in detail by our group.^20^ To a soln of 10-BrPhEtyCbl (10.8 mg, 7.16 μmol, 1.0 equiv.) in H_2_O (2.0 mL), a soln of HBF_4_ (48% in H_2_O, 100 μL, 34.3 mg HBF_4_, 390 μmol) was added and the resultant bright purple soln was stirred at 40 °C for 10 min. LC-MS analysis (method 1) of the reaction mixture revealed successful de-alkynylation of the starting material, yielding 6 as the sole product, which was subsequently isolated from the reaction mixture using SPE, eluted with CH_3_OH and the solvent was removed *in vacuo* (200 mbar, 40 °C). The residue was re-dissolved in H_2_O and lyophilized overnight to yield 6 (10.5 mg, 6.94 μmol, 97%).

UV/vis (H_2_O, *c* = 1.1 × 10^−5^ M): 280 (4.2), 289 (4.2), 357 (4.4), 421 (3.6), 532 (3.9), 555 (3.9). UPLC: *t*_ret_ = 1.75 min (method 1). ESI-MS (H_2_O/MeCN): *m*/*z* = 704.81 (100, [M–H_2_O + 2H]^2+^, *m*/*z*_calc_: 704.74 for C_62_H_88_BrCoN_13_O_14_P^2+^). ^1^H-NMR (D_2_O, *c* = 1.1 × 10^−3^ M): 7.08 (s, *H*C7N), 6.44 (s, *H*C2N), 6.35 (s, *H*C4N), 6.16–6.14 (d-like m, *H*C1R), 4.23–4.10 (m, *H*C19, *H*C176, *H*C2R, *H*C8), 3.91 (d, *J* = 8.3, *H*C4R), 3.79 (d, *J* = 12.0, H_a_ of H_2_C5R), 3.62 (d, *J* = 12.0, H_b_ of H_2_C5R), 3.56–3.46 (m, H_a_ of C175, *H*C13), 2.90–2.77 (m, H_b_ of H_2_C175, *H*C18), 2.71–2.38 (m, *H*_2_C181, *H*_2_C132 H_a_ of H_2_C171, *H*_2_C172) superimposed by 2.60 (s, H_3_C151) and 2.54 (s, H_3_C51), 2.36–2.27 (m, *H*_2_C32, H_a_ of H_2_C71), 2.22–1.69 (m, *H*_2_C21, *H*_2_C31, H_a_ of H_2_C81, H_b_ of H_2_C171, *H*_2_C172, *H*_2_C71) superimposed by 2.17 (s, *H*_3_C10N), 2.13 (s, *H*_3_C11N), 1.87 (s, *H*_3_C7A) and 1.75 (s, *H*_3_C12A), 1.42–1.10 (m, H_b_ of H_2_C81, *H*_2_C82) superimposed by 1.38 (s, *H*_3_C2A), 1.33 (s, *H*_3_C17B), 1.29 (s, *H*_3_C12B) and 1.15 (d, *J* = 6.0, *H*_3_C177), 0.31 (s, *H*_3_C1A). Chemical shifts were identical with those published earlier.^20^

#### 10-Bromo-Co_β_-phenylcobalamin (7)

To a soln of 8 (10 mg, 7.1 μmol, 1.0 equiv.) in conc. acetic acid (0.5 mL), NBS (1.3 mg, 7.1 μmol, 1.0 equiv.) was added in small portions over a time period of 90 min at 23 °C under protection from light. The soln turned purple and was diluted with 0.1 M Tris buffer (pH = 8.0, 10 mL) and the raw product (no formation of Co_α/β_ diastereomers observed) was extracted using SPE. Purification *via* prep. HPLC (method B) and crystallization from H_2_O/MeCN afforded 7 (5.4 mg, 3.6 μmol, 51%) as bright purple needles.

UV-vis (H_2_O, *c* = 3.5 × 10^−5^ M): 284 (4.04), 348 (4.00), 380 (sh., 3.79), 471 (sh., 3.47), 537 (br., 3.67). UPLC: *t*_ret_ = 2.65 min (method 1). HRMS (ESI+): *m*/*z* = 742.76424 (100, [C_68_H_92_O_14_N_13_BrCoP + 2H]^2+^, *m*/*z*_calc_: 742.76481), *m*/*z* = 1484.52168 (30, [M + H]^+^, *m*/*z*_calc_ = 1484.52125). ^1^H-NMR (CD_3_OD, *c* = 8.7 × 10^−3^ M): *δ* 8.30 (bs, *H*C2N), 7.41 (s, *H*C7N), 7.17 (s, *H*C4N), 6.74 (t, *J* = 7.0 Hz, *H*C4L), 6.60 (t, *J* = 7.6 Hz, *H*C3L–*H*C5L), 6.34 (d, *J* = 4.2 Hz, *H*C1R), 5.32 (d, *J* = 7.3 Hz, *H*C2L–*H*C6L), 4.62 (dd, *J* = 2.8, 7.5 Hz, *H*C3R), 4.53 (t-like, *H*C2R), 4.45–4.38 (m, *H*C4R), 4.38–4.30 (m, *H*C176), 3.88 (dd, *J* = 12.4, 3.1 Hz, H_a_ of H_2_C5R), 3.76 (dd, *J* = 12.4, 4.1 Hz, H_b_ of H_2_C5R), 3.57–3.46 (m, H_a_ of H_2_C175, *H*C13, *H*C19), 3.05 (dd, *J* = 13.9, 7.5 Hz, H_b_ of H_2_C175), 3.02–2.96 (m, *H*C18), 2.74 (s, *H*_3_C51), 2.70–2.66 (d, H_a_ of H_2_C71) superimposed by 2.67 (s, *H*_3_C151), 2.65–2.53 (m, H_a_ of H_2_C171, *H*_2_C132), 2.49–2.33 (m, *H*_2_C32, corrin-C*H*_2_, H_a_ of H_2_C172) superimposed by 2.40 (s, *H*_3_C10N) and 2.37 (s, *H*_3_C11N), 2.28–2.14 (m, H_b_ of H_2_C171, H_a_ of H_2_C82, H_a_ of corrin-C*H*_2_, H_b_ of H_2_C172), 2.10–2.00 (m, *H*_2_C31, H_b_ of H_2_C82), 1.95 (d, H_a_ of H_2_C21) superimposed by 1.96 (s, *H*_3_C7A), 1.90–1.87 (d, H_b_ of H_2_C21) superimposed by 1.87 (s, *H*_3_C12A), 1.79–1.67 (m, H_b_ of corrin), 1.64 (d, *J* = 14.1 Hz, H_b_ of H_2_C71), 1.35 (s, *H*_3_C2A), 1.23 (d, *J* = 6.2 Hz, *H*_3_C177), 1.18 (s, *H*_3_C17B), 1.14 (s, *H*_3_C12B), 0.90 (s, *H*_3_C1A). ^13^C-NMR (CD_3_OD, *c* = 8.7 × 10^−3^ M): *δ* 178.1, 177.9, 177.6, 177.5, 176.9, 175.9, 175.4, 174.9, 174.6, 173.0, 165.9, 164.0, 142.6 (C2N), 135.4, 134.1 (C2L, C6L), 131.8, 130.3, 128.2 (C3L, C5L), 126.3 (C4L), 118.0 (C4N), 112.9 (C7N), 111.2, 107.9, 94.2 (C10), 88.2, 87.6 (C1R), 85.7, 77.2 (C19), 75.8 (C3R), 74.5, 73.1, 72.3, 62.8, 60.9, 58.2, 57.8, 57.7, 57.5, 57.3, 57.1 (C3), 57.1 (C13), 52.2, 47.1, 46.5 (C175), 43.5 (C71), 42.4 (C21), 39.8 (C18), 36.4 (C32), 34.4, 34.3 (C132), 34.2 (C172), 33.4, 33.0 (C171), 28.9, 28.7 (C82), 27.8 (C12B), 26.9 (C31), 23.9 (C12A), 23.6 (C1A), 20.7 (C10N), 20.5 (C11N), 20.0 (C177), 19.8 (C7A), 19.1 (C17B), 17.3 (C51), 17.2 (C2A), 17.0 (C151). Assignments were made based on 2D NMR studies (HSQC and HMBC) and comparison with data of 4.

#### Co_β_-phenylcobalamin (8; β-PhCbl)

was synthesized using a modified literature procedure.^13^ In a Schlenk tube, Co_β_-aquacobalamin chloride (25.0 mg, 18.1 μmol, 1 equiv.) was dissolved in H_2_O (2.0 mL). After degassing by pump–freeze–thaw cycling (three cycles), sodium borohydride (6.90 mg, 180 μmol, 10 equiv.) was added under N_2_ counterflow. Upon addition, the solution turned dark brown, and the mixture was stirred at 23 °C for 30 min, before diphenyliodonium chloride (11.5 mg, 37.0 μmol, 2.0 equiv.) was added. The solution was protected from light and stirred at 23 °C for further 120 min. The reaction mixture was diluted with H_2_O (10 mL) and the raw products were extracted using SPE. The solvent was evaporated under reduced pressure and the residue was purified *via* preparative HPLC (method A). Isolated 8 was transferred to its base-on form by treatment with 10% aq. NaOH, followed by washing with water. Crystallization from aqueous acetone furnished 8 (15.0 mg, 12 μmol, 59%) as a dark red crystalline solid. Co_α_-phenylcobalamin was not isolated.

UV-vis (H_2_O, *c* = 6.4 × 10^−5^ M): 267 (3.95), 283 (3.93), 342 (3.83), 374 (3.69), 475 (sh., 3.42), 520 (br., 3.58). UPLC: *t*_ret_ = 2.52 min (method 1). ESI-MS (H_2_O/MeCN): *m*/*z* = 703.96 (100, [M + 2H]^2+^), 1406.63 (7, [M + H]^+^, *m*/*z*_calc_: 1406.61 for C_68_H_94_CoN_13_O_14_P^+^). ^1^H-NMR (D_2_O, *c* = 3.2 × 10^−5^ M): 7.22 (s, *H*C2N), 7.16 (s, *H*C7N), 6.79 (t, *J* = 6.7, *H*C4L), 6.74 (t, *J* = 7.3, *H*C3L, *H*C5L), 6.59 (s, *H*C4N), 6.23 (d, *J* = 3.0, *H*C1R), 5.96 (s, HC10), 5.83 (d, *J* = 7.5, *H*C2L, *H*C6L), 4.69–4.64 (m, *H*C3R), 4.30 (d, *J* = 7.5, HC176), 4.24–4.21 (m, *H*C3, *H*C2R), 4.11–4.09 (m, *H*C4R), 3.92 (app. d, *J* = 11.3, H_a_ of H_2_C5R), 3.73 (dd, *J* = 12.8, 3.8, H_b_ of H_2_C5R), 3.52 (app. d, *J* = 14.3, H_a_ of H_2_C175), 3.41–3.32 (m, 3 corrin-C*H*), 2.96 (dd, *J* = 14.7, 8.7 Hz, H_b_ of H_2_C175), 2.73–2.56 (m, corrin-C*H*, 2 corrin-C*H*_2_) superimposed by 2.73 (s, *H*_3_C51) and 2.63 (s, *H*_3_C151), 2.49–2.40 (m, 2 corrin-C*H*_2_), 2.28 (s, *H*_3_C10N), 2.22 (s, *H*_3_C11N), 2.15–2.03 (m, 2 corrin-C*H*_2_), 1.96–1.93 (m, corrin-C*H*_2_), 1.89–1.63 (m, 4 corrin-C*H*_2_) superimposed by 1.85 (s, *H*_3_C7A), 1.40 (s, *H*_3_C12A), 1.24 (s, *H*_3_C2A), 1.21–1.09 (m, H_b_ of H_2_C82) superimposed by 1.19 (d, *J* = 6.8, *H*_3_C177) and 1.12 (s, *H*_3_C17B), 0.97 (d, *J* = 9.8, H_b_ of H_2_C81), 0.90 (s, *H*_3_C12B), 0.47 (s, *H*_3_C1A). Assignments were made in comparison with data from lit.^13^

## Author contributions

F. Z. and C. B. designed the experiments and wrote the manuscript. C. B. and P. D. M. performed synthesis and characterization. C. B. executed the biological studies.

## Conflicts of interest

There are no conflicts to declare.

## Supplementary Material

RA-012-D2RA05748D-s001
