# Supplementary material for: Functionalisation of vitamin B12 derivatives with a cobalt β-phenyl ligand boosters antimetabolite activity in bacteria
Source: RSC Adv. 2022 Oct 7;12(44):28553–9. doi: 10.1039/d2ra05748d (PMC9541496; doi:10.1039/d2ra05748d)
Supplement: RA-012-D2RA05748D-s001 [file RA-012-D2RA05748D-s001.pdf]

Electronic Supplementary Material (ESI) for RSC Advances.
This journal is © The Royal Society of Chemistry 2022

Supporting Information

Functionalisation of Vitamin B_{12} Derivatives with a Cobalt β-Phenyl Ligand Boosters Antimetabolite Activity in Bacteria

Dr. Christopher Brenig, Paula Daniela Mestizo and Prof. Dr. Felix Zelder[*]

Department of Chemistry, University of Zurich, Winterthurerstrasse 190, CH-8057 Zurich, Switzerland. Fax: +41 44 635 6803; E-mail: felix.zelder@chem.uzh.ch, www.felix-zelder.net.

Contents

Reaction Schemes. S4

- **Scheme S1** S4
  Synthesis of Co$_\beta$-cyanocobalamin-*c*,8-lactam (**2**)
- **Scheme S2** S4
  Synthesis of *Co$_\beta$*-aqua-cobalamin-*c,*8-lactam acetate (**3**)
- **Scheme S3** S4
  Synthesis of Co$_\beta$-phenylcobalamin-*c*,8-lactam (**4**)
- **Scheme S4** S5
  Synthesis of 10-bromo-Co$_\beta$-cyanocobalamin (**5**)
- **Scheme S5** S5
  Synthesis of 10-bromo-Co$_\beta$-aquacobalamin tetrafluoroborate (**6**)
- **Scheme S6** S5
  Synthesis of 10-bromo-Co$_\beta$-phenylcobalamin (**7**)

Atom Numbering. S6

- **Figure S1** S6
  Atom numbering of vitamin B_{12} (**1**) and its analogues

NMR and optical Spectra. S7

- **Figure S2** S7
  ^{1}H-NMR spectrum of **2** (500 MHz, D_2O (presat), 298 K, 4.1 x 10^{-3} M).
- **Figure S3** S7
  UV-Vis spectrum of **2** (H_2O, 4.1 x 10^{-5} M).
- **Figure S4** S8
- ^{1}H-NMR spectrum of **3** (500 MHz, CD_3OD, 298 K, 7.3 · 10^{-3} M).
- **Figure S5** S8
  UV/Vis spectrum of **3** (H_2O, c = 2.2 · 10^{-5} M).
- **Figure S6** S9
  ^{1}H-NMR spectrum of **4** (500 MHz, D_2O (presat), 298 K, 1.2 x 10^{-2} M).
- **Figure S7** S9
  ^{13}C-NMR spectrum of **4** (126 MHz, D_2O (presat), 298 K, 1.2 x 10^{-2} M).
- **Figure S8** S10
  DEPT 135-NMR spectrum of **4** (126 MHz, D_2O, 298 K, 1.2 x 10^{-2} M).
- **Figure S9** S10
  ^{1}H-^{1}H-DFQ-COSY spectrum of **4** (500 MHz, D_2O, 298 K, 1024 x 2560 p, 12 scans, 1.2 x 10^{-2} M).
- **Figure S10** S11
  ^{1}H-^{1}H-NOESY spectrum of **4** (500 MHz, D_2O, 298 K, 1024 x 1884 p, 16 scans, 1.2 x 10^{-2} M).
- **Figure S11** S11
  ^{1}H-^{13}C-HSQC spectrum of **4** (500 MHz, 126 MHz, D_2O (presat), 298 K, 1024 x 5120 p, 18 scans, 1.2 x 10^{-2} M).
- **Figure S12** S12
  ^{1}H-^{13}C-HMBC spectrum of **4** (500 MHz, 126 MHz, D_2O, 298 K, 1024 x 3000 p, 12 scans, 1.2 x 10^{-2} M).

  Figure S13 S12
  UV/Vis spectrum of **4** (H_2O, 3.9 x 10^{-5} M).
- **Figure S14** S13
  ^{1}H-NMR spectrum of **5** (500 MHz, D_2O (presat), 298 K, 4.9 x 10^{-3} M).
- Figure S15 **S13**
  UV-Vis spectrum of **5** (H_2O, 4.9 x 10^{-5} M).
- **Figure S16** S14
  ^{1}H-NMR spectrum of **7** (500 MHz, CD_3OD (presat), 298 K, 8.7 x 10^{-3} M).
- **Figure S17** S14
  ^{13}C-NMR spectrum of **7** (126 MHz, CD_3OD (presat), 298 K, 8.7 x 10^{-3} M).
- **Figure S18** S15
  ^{1}H-^{13}C-HSQC spectrum of **7** (500 MHz, 126 MHz, CD_3OD, 298 K, 1024 x 1024 p, 32 scans, 8.7 x 10^{-3} M).
- **Figure S19** S15
  ^{1}H-^{13}C-HMBC spectrum of **7** (500 MHz, 126 MHz, CD_3OD, 298 K, 1024 x 2341 p, 12 scans, 8.7 x 10^{-3} M).
- **Figure S20** S16
  UV/Vis spectrum of **7** (H_2O, 3.5 x 10^{-5} M).
- **Figure S21** S16
  ^{1}H-NMR spectrum of **8**, **β-PhCbl** (500 MHz, D_2O (presat), 298 K, 3.2 x 10^{-3} M).

- **Figure S22** S17

^{1}H-NMR spectrum of **8**, **β-PhCbl** (500 MHz, D_2O (presat), 298 K, 3.2 x 10^{-3} M).

Reaction Schemes.

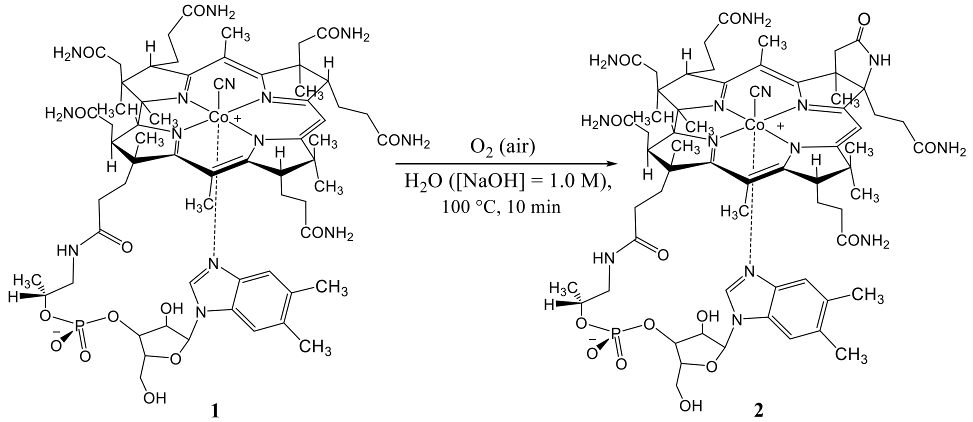

1
$C_{63}H_{88}CoN_{14}O_{14}P$
[1355.39]

2
$C_{63}H_{86}CoN_{14}O_{14}P$
[1353.37]

Scheme S1. Synthesis of Co$_\beta$-cyanocobalamin-*c*,8-lactam (**2**).

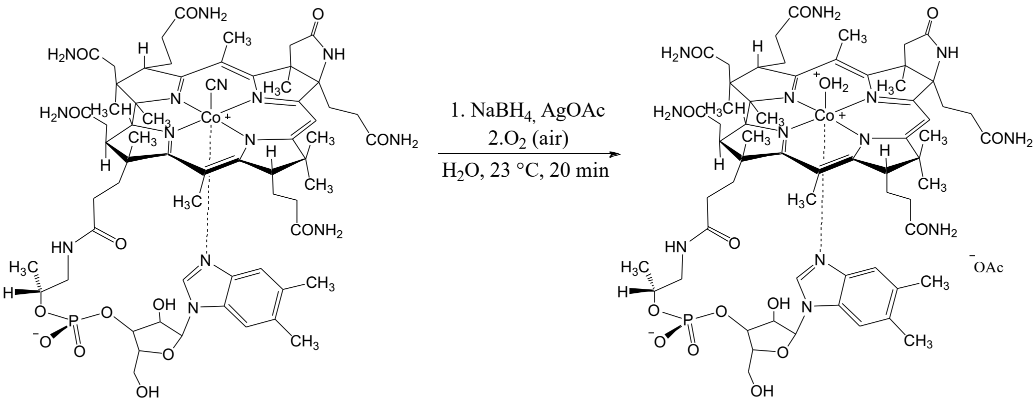

2
$C_{63}H_{86}CoN_{14}O_{14}P$
[1353.37]

3
$C_{64}H_{91}CoN_{13}O_{17}P$
[1404.41]

Scheme S2. Synthesis of *Co$_\beta$*-aquacobalamin-*c,*8-lactam acetate (**3**)

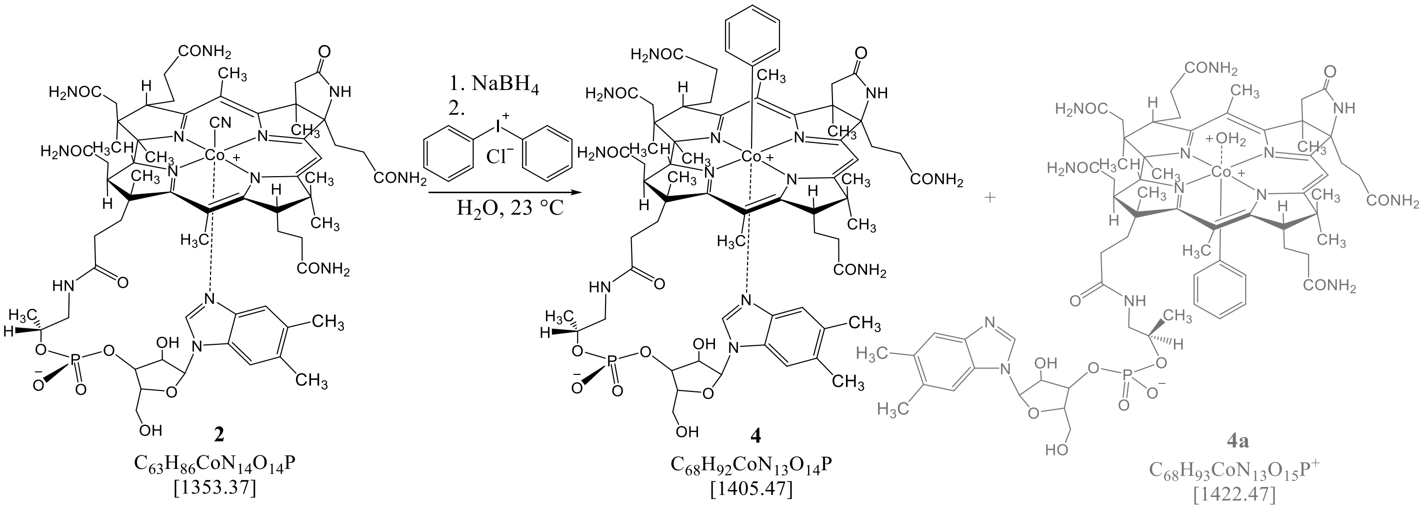

2
$C_{63}H_{86}CoN_{14}O_{14}P$
[1353.37]

4
$C_{68}H_{92}CoN_{13}O_{14}P$
[1405.47]

4a
$C_{68}H_{93}CoN_{13}O_{15}P^+$
[1422.47]

Scheme S3. Synthesis of Co$_\beta$-phenylcobalamin-*c*,8-lactam (**4**) and its side product Co$_\alpha$-phenylcobalamin-*c*,8-lactam (**4a,** not characterized).

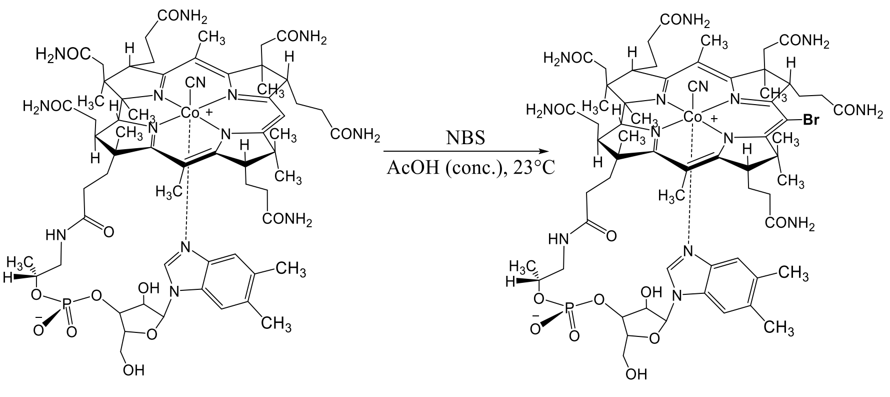

1
$C_{63}H_{88}CoN_{14}O_{14}P$
[1355.39]

5
$C_{63}H_{87}BrCoN_{14}O_{14}P$
[1434.28]

Scheme S4. Synthesis of 10-bromo-Coβ-cyanocobalamin (**5**).

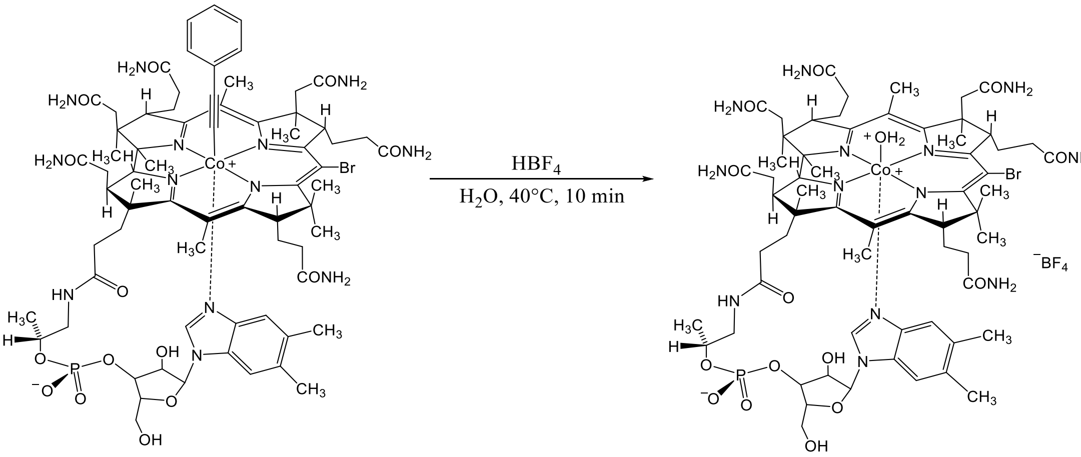

10-BrPhEtyCbl
$C_{70}H_{92}BrCoN_{13}O_{14}P$
[1509.39]

6
$C_{62}H_{89}BBrCoF_4N_{13}O_{15}P$
[1513.08]

Scheme S5. Synthesis of 10-bromo-Coβ-aquacobalamin tetrafluoroborate (**6**) from intermediate 10-Bromo- Coβ-phenylethynylcobalamin (reported in ref[S4]).

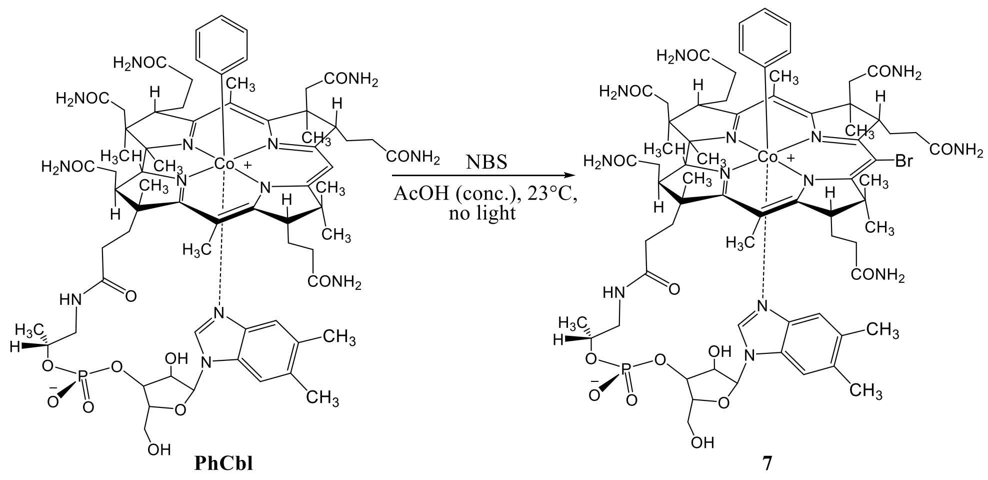

PhCbl
$C_{68}H_{93}CoN_{13}O_{14}P$
[1406.48]

7
$C_{68}H_{92}BrCoN_{13}O_{14}P$
[1485.37]

Scheme S6. Synthesis of 10-bromo-Coβ-phenylcobalamin (**7**) from Coβ-phenylcobalamin (reported in ref[S3]).

Atom Numbering.

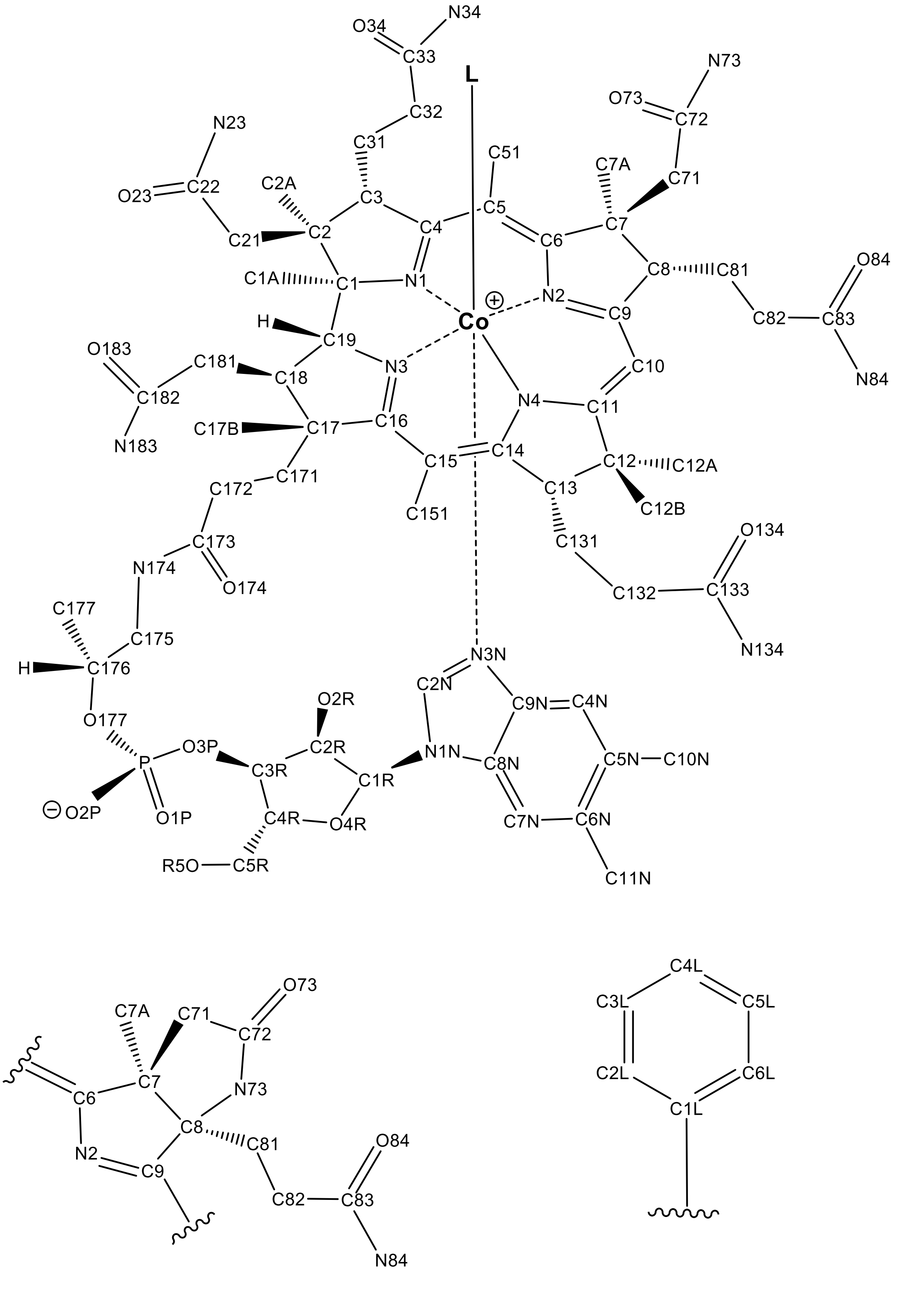

Figure S1. Atom numbering of vitamin B_{12} (**1**) and its analogues. *Bottom*: Atom numbering of the *c*,8-lactam ring in derivatives **2** and **4**, as well as nomenclature of the phenyl ligand of **β-PhCbl**, **4** and **7**.

NMR and optical Spectra.

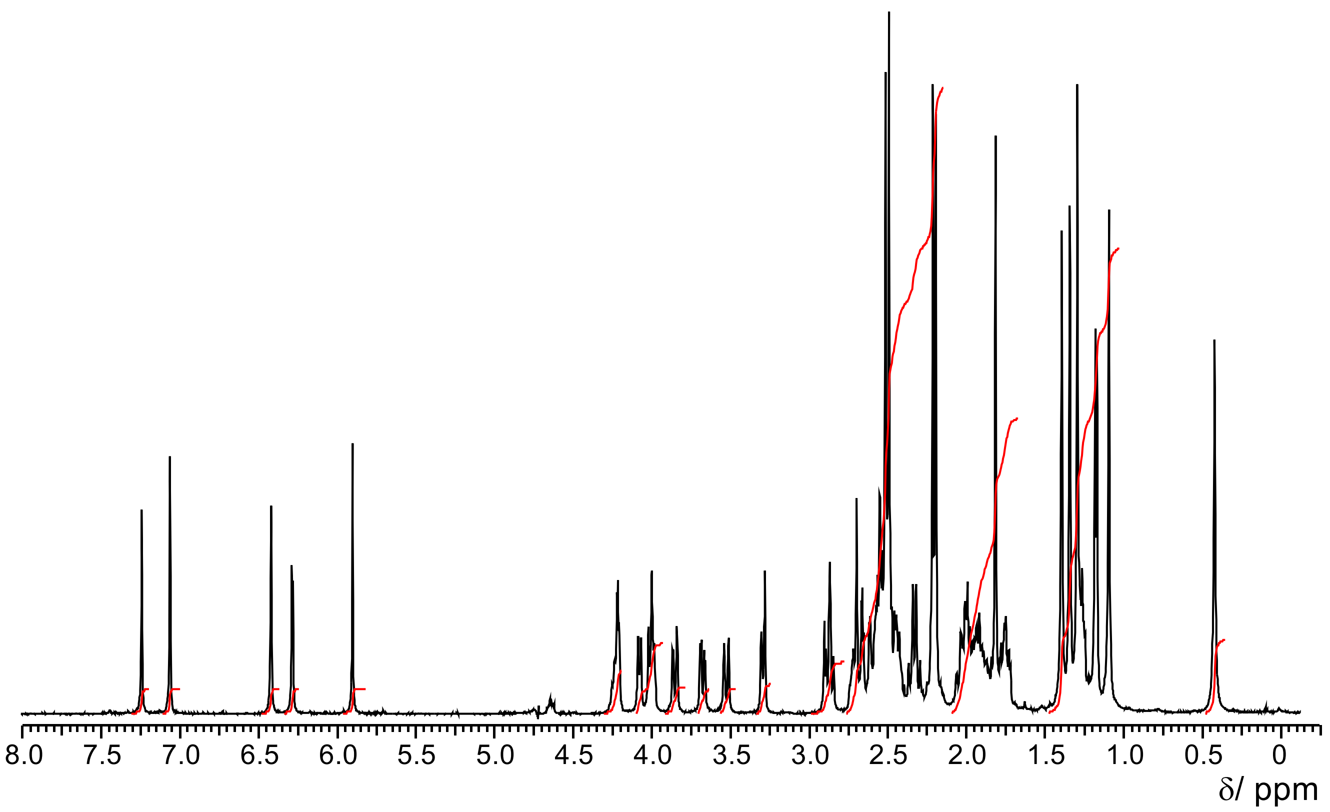

Figure S2. ^{1}H-NMR spectrum of **2** (500 MHz, D_2O (presat), 298 K, 4.1 x 10^{-3} M).

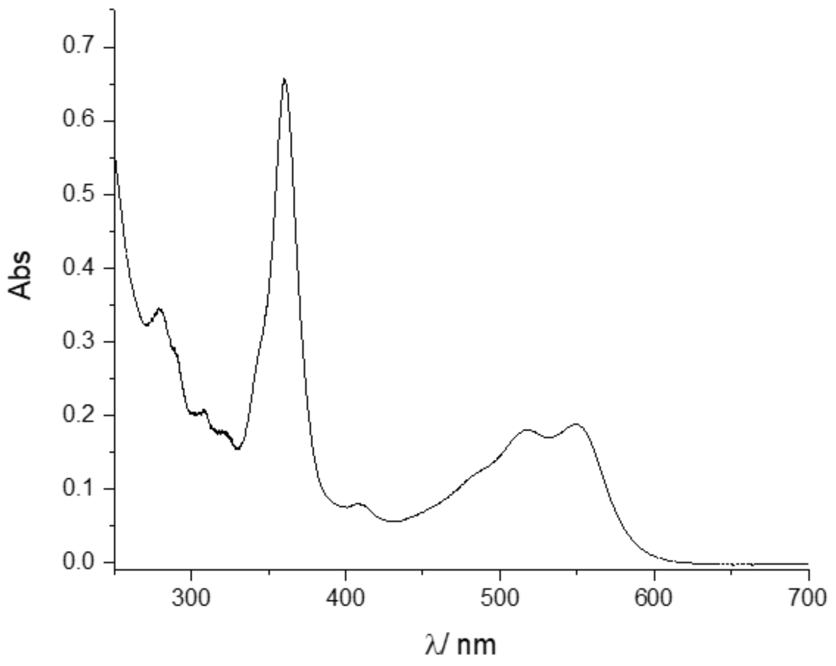

Figure S3. UV/Vis spectrum of **2** (H_2O, 4.1 x 10^{-5} M).

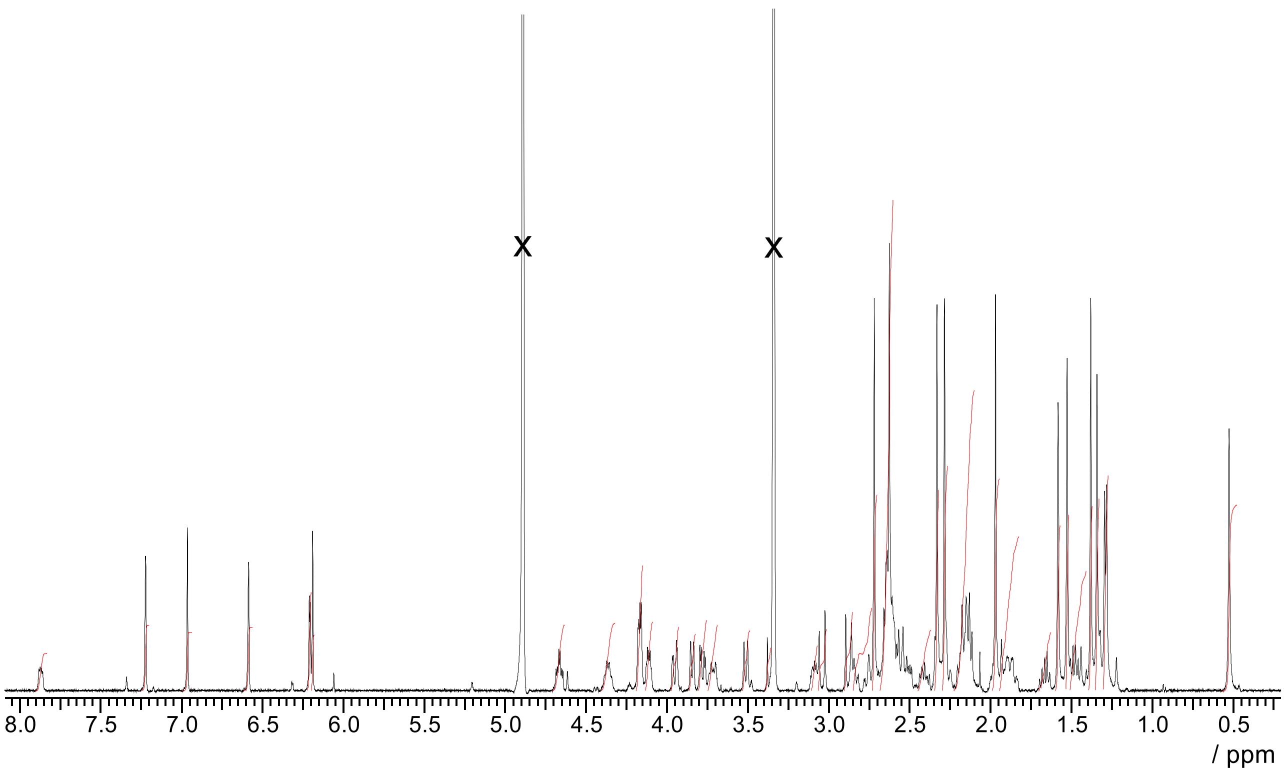

Figure S4. ^{1}H-NMR spectrum of **3** (500 MHz, CD_3OD, 298 K, $7.3 \cdot 10^{-3}$ M).

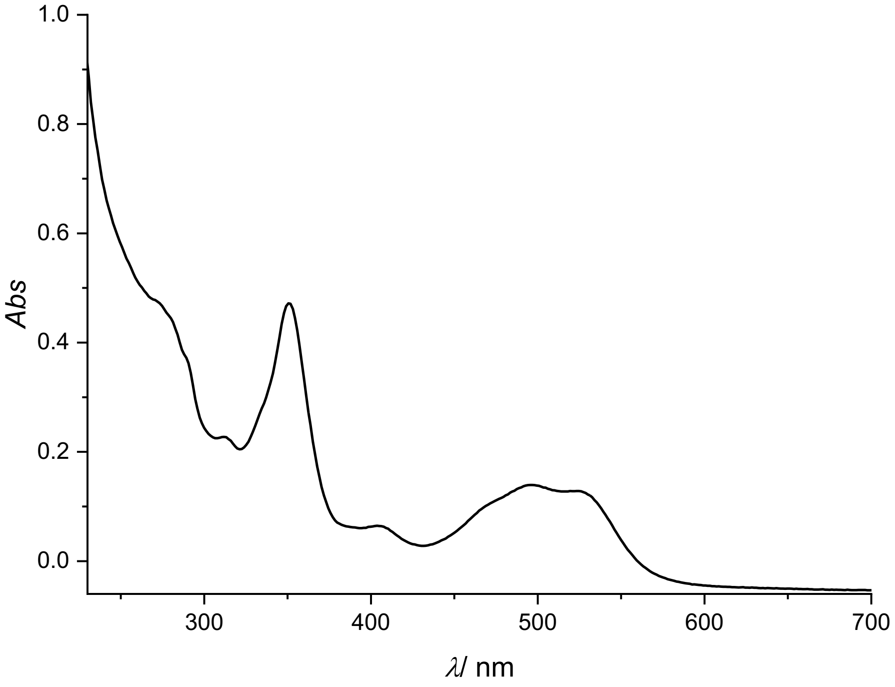

Figure S5. UV/Vis spectrum of **3** (H_2O, c = $2.2 \cdot 10^{-5}$ M).

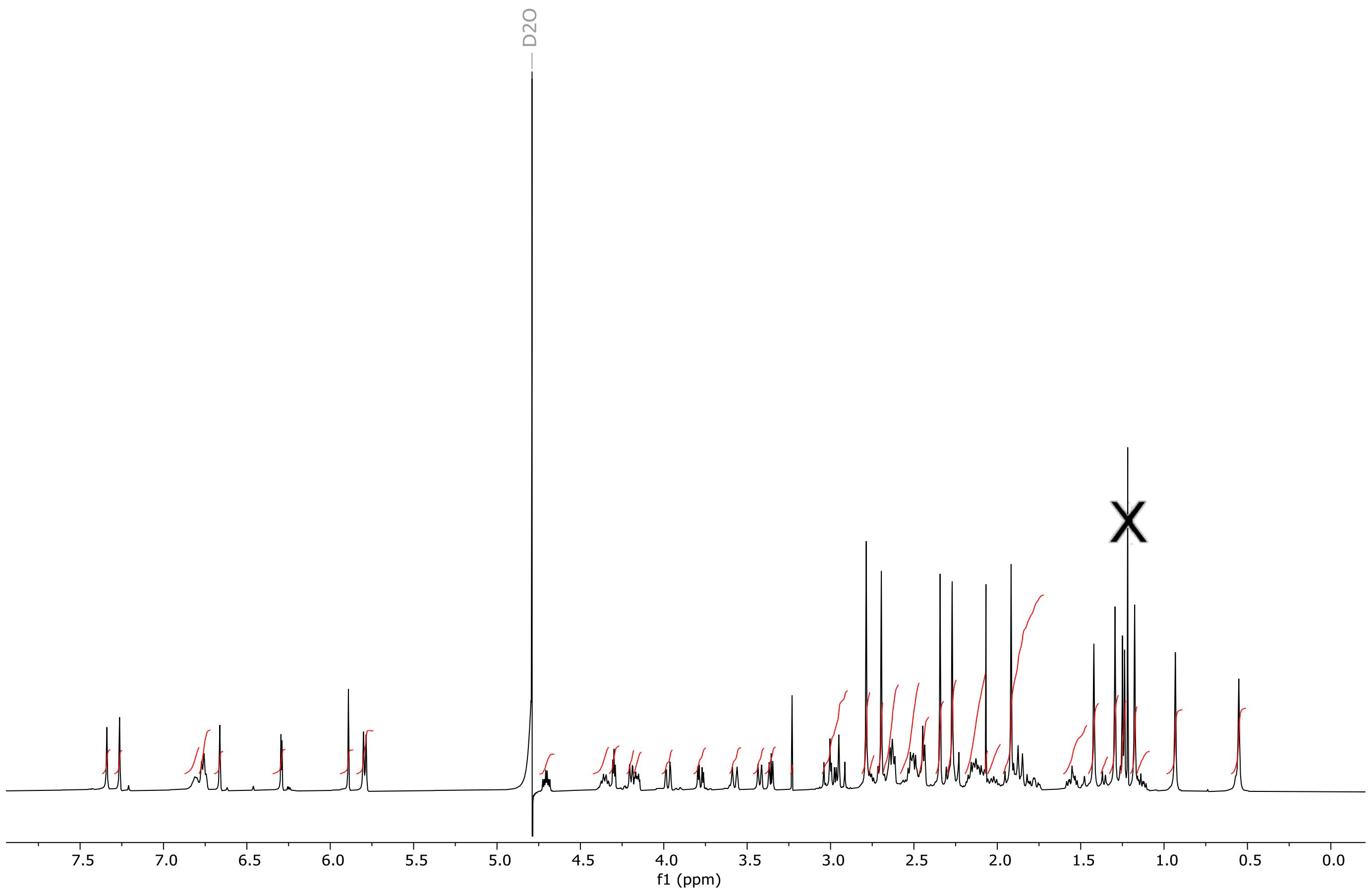

Figure S6. ^{1}H-NMR spectrum of **4** (500 MHz, D_2O (presat), 298 K, 1.2 x 10^{-2} M).

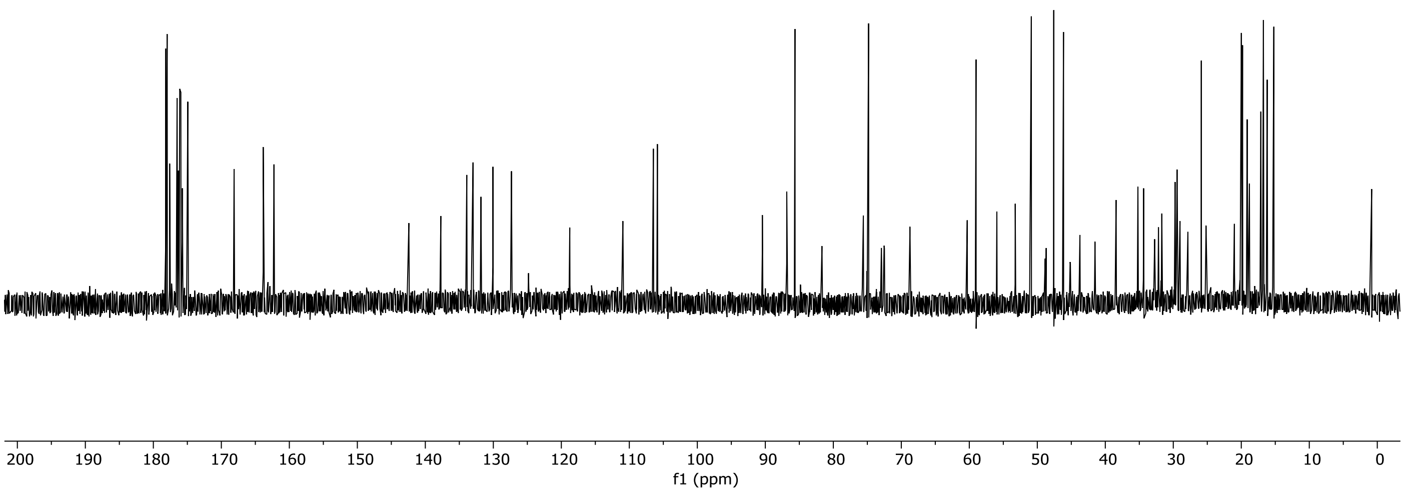

Figure S7. ^{13}C-NMR spectrum of **4** (126 MHz, D_2O, 298 K, 1.2 x 10^{-2} M).

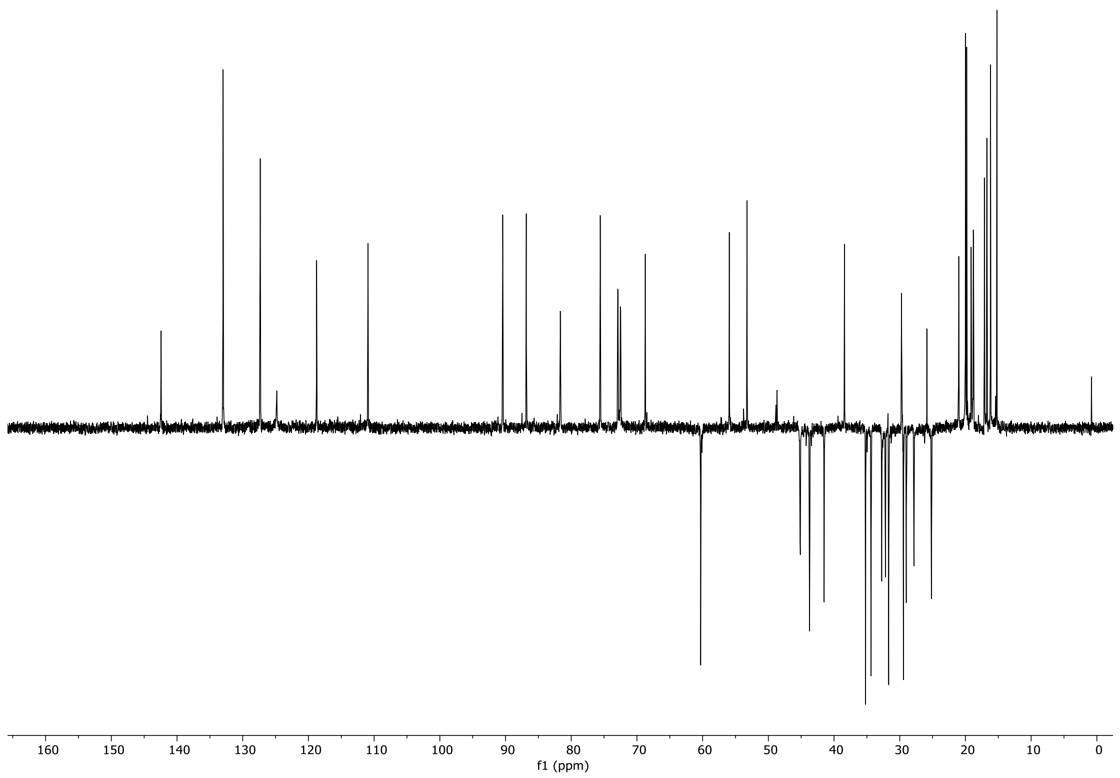

Figure S8. DEPT 135-NMR spectrum of **4** (126 MHz, D_2O, 298 K, 1.2 x 10^{-2} M).

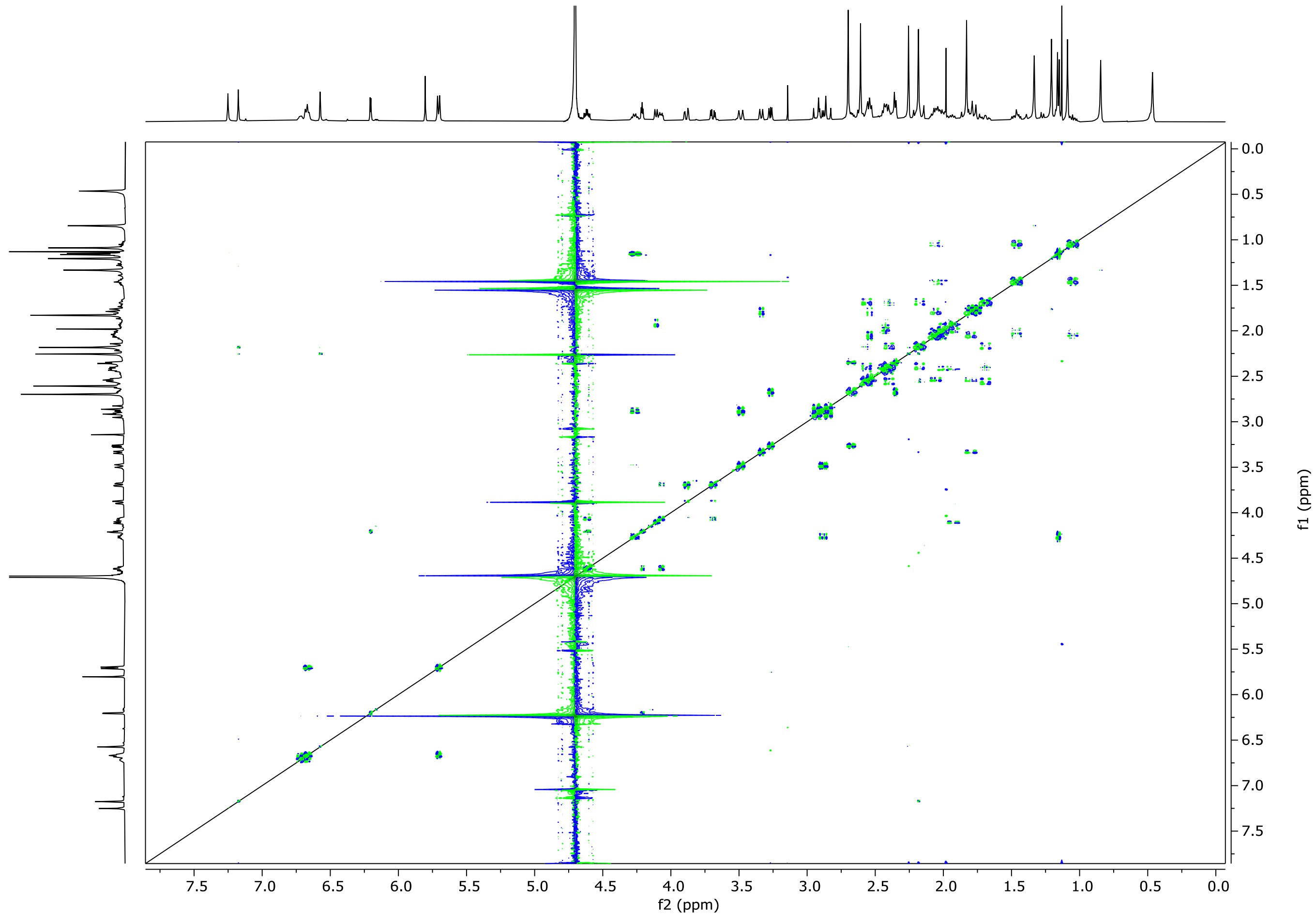

Figure S9. ^1H-^1H DFQ-COSY spectrum of **4** (500 MHz, D_2O, 298 K, 1024 x 2560 p, 12 scans, 1.2 x 10^{-2} M).

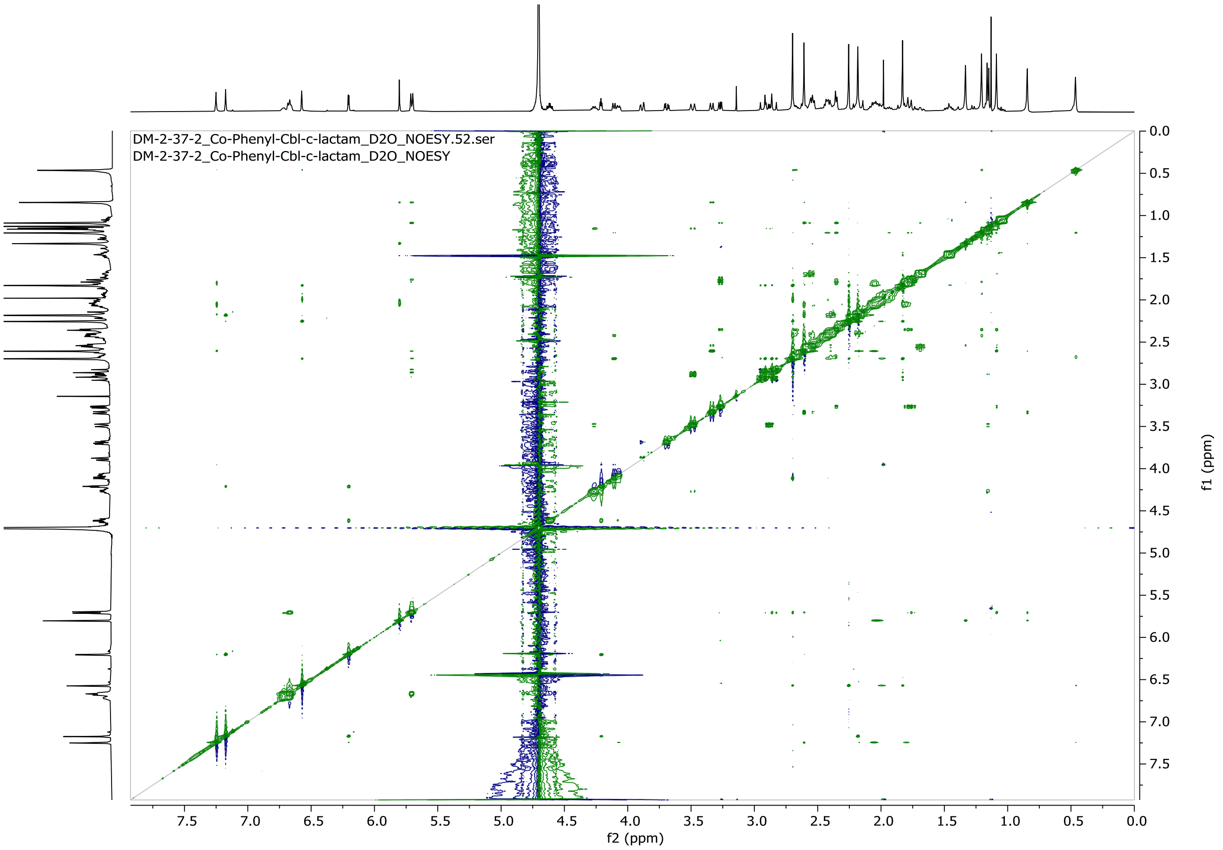

Figure S10. ^{1}H-^{1}H-NOESY spectrum of **4** (500 MHz, D_2O, 298 K, 1024 x 1884 p, 16 scans, 1.2 x 10^{-2} M).

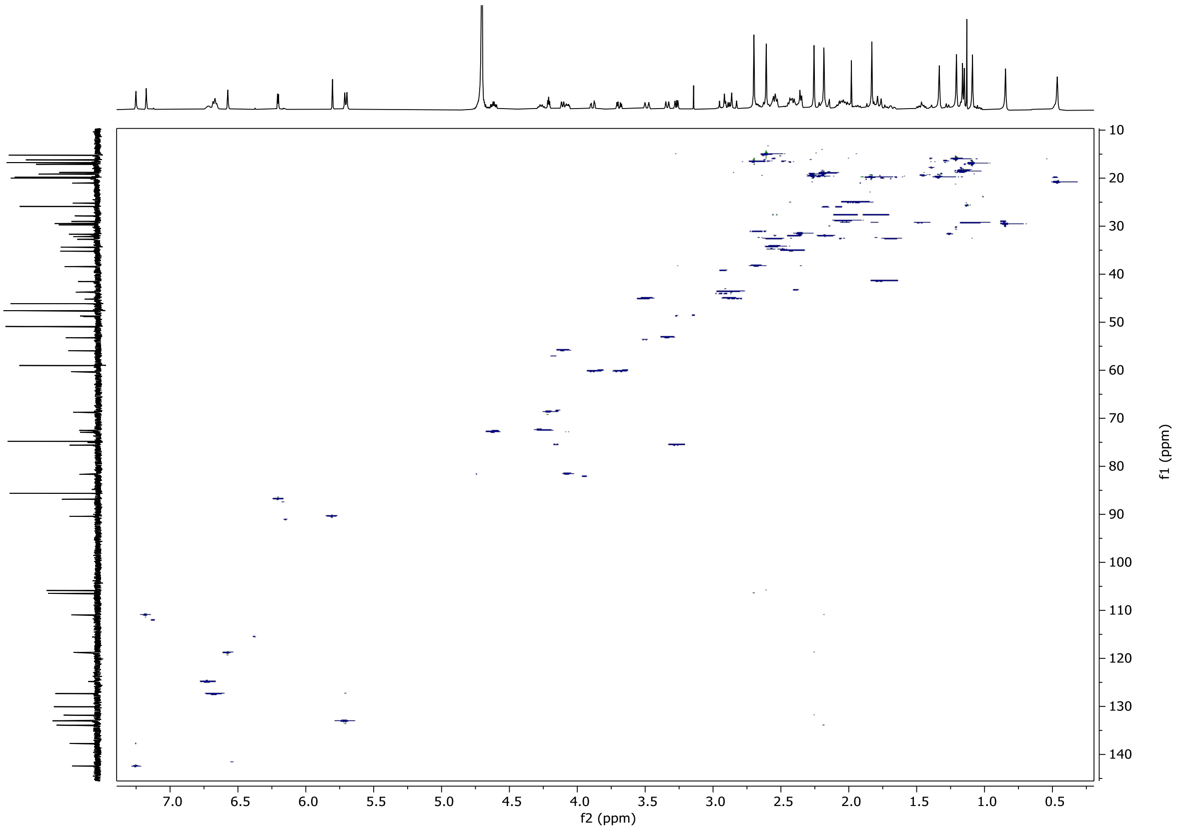

Figure S11. ^{1}H-^{13}C-HSQC spectrum of **4** (500 MHz, 126 MHz, D_2O (presat), 298 K, 1024 x 5120 p, 18 scans, 1.2 x 10^{-2} M).

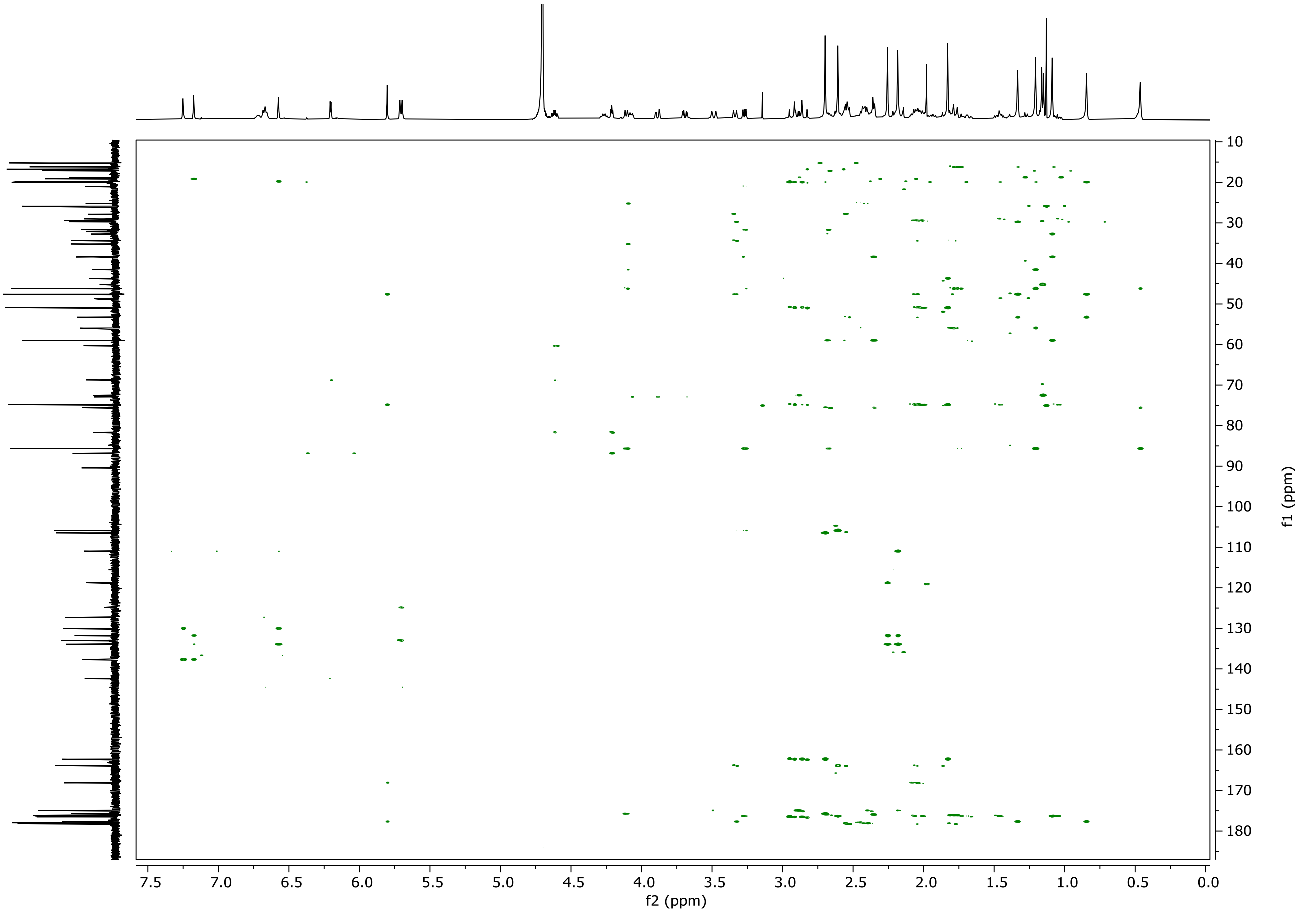

Figure S12. ^{1}H-^{13}C-HMBC spectrum of **4** (500 MHz, 126 MHz, D_2O, 298 K, 1024 x 3000 p, 12 scans, 1.2 x 10^{-2} M).

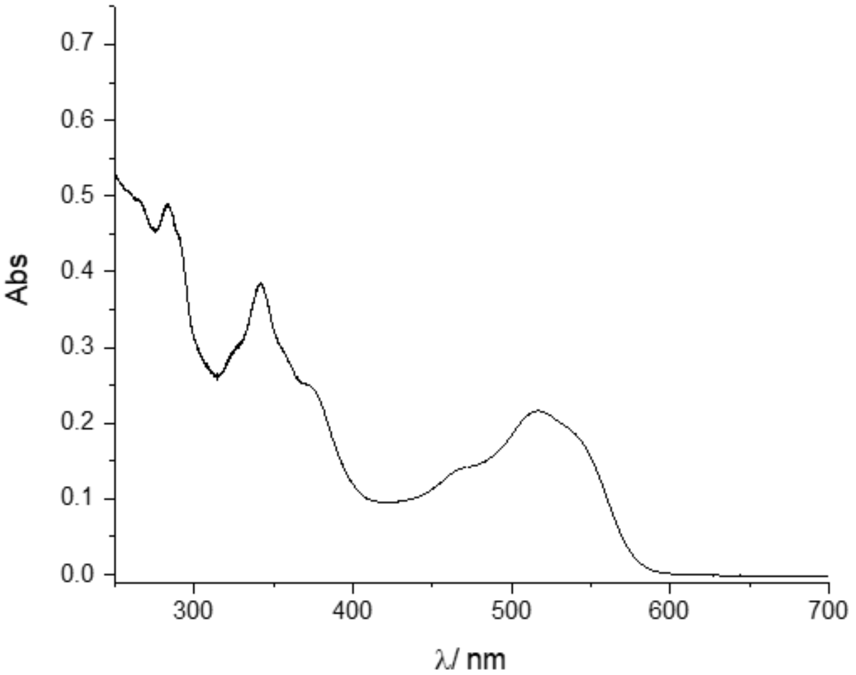

Figure S13. UV/Vis spectrum of **4** (H_2O, 3.9 x 10^{-5} M).

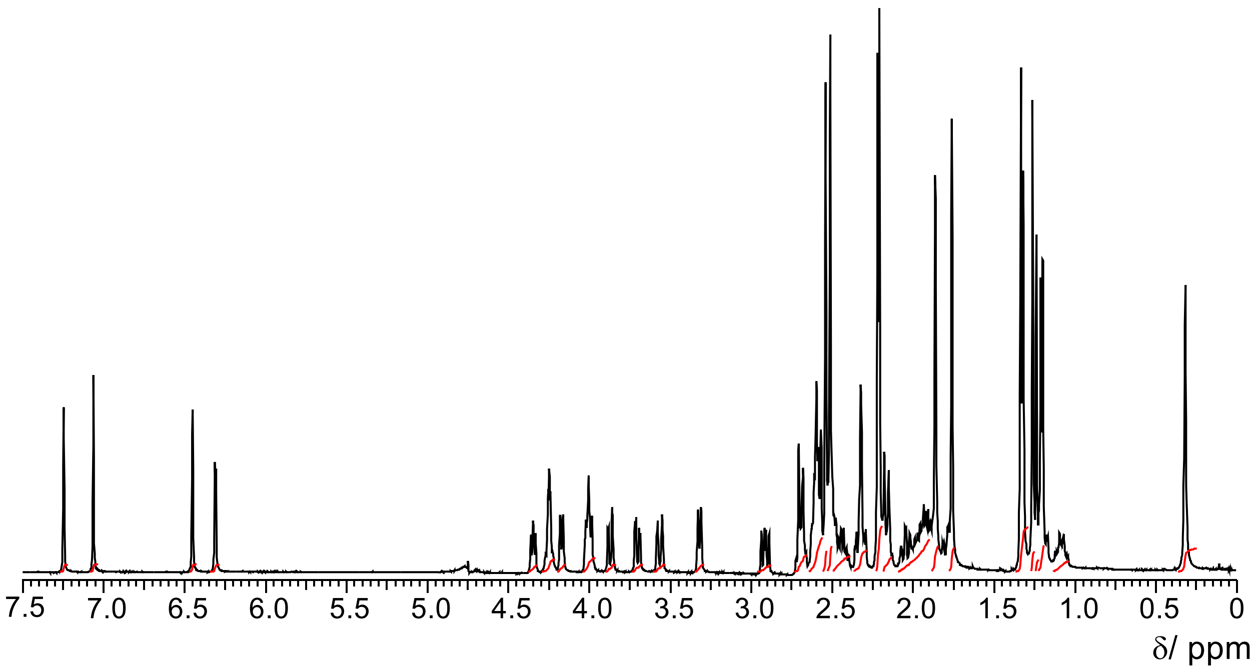

Figure S14. ^{1}H-NMR spectrum of **5** (500 MHz, D_2O (presat), 298 K, 4.9 x 10^{-3} M).

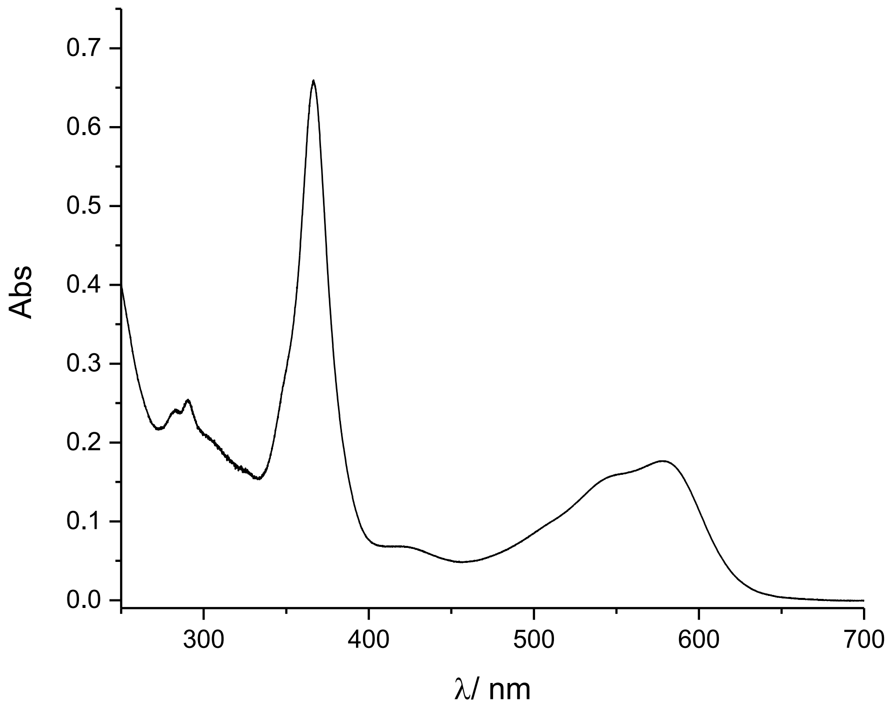

Figure S15. UV/Vis spectrum of **5** (H_2O, 4.9 x 10^{-5} M).

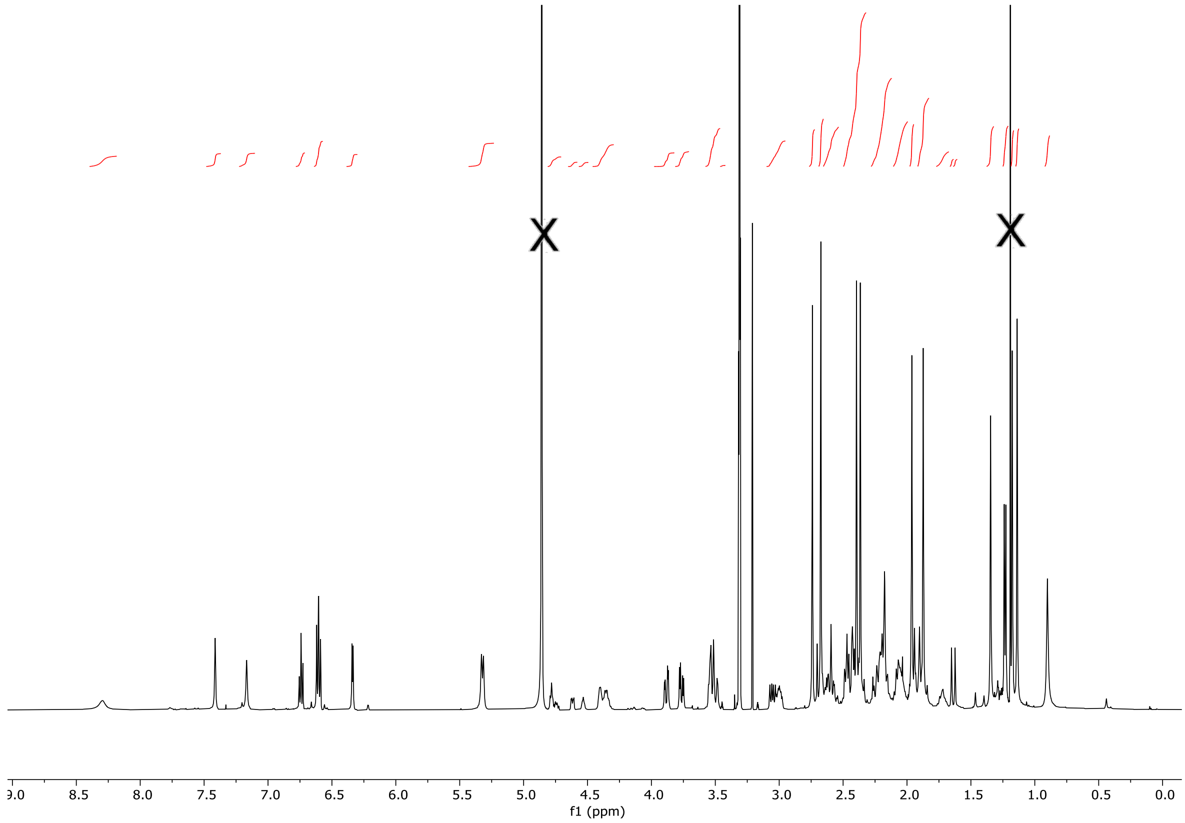

Figure S16. ^{1}H-NMR spectrum of **7** (500 MHz, CD_3OD (presat), 298 K, 8.7 x 10^{-3} M).

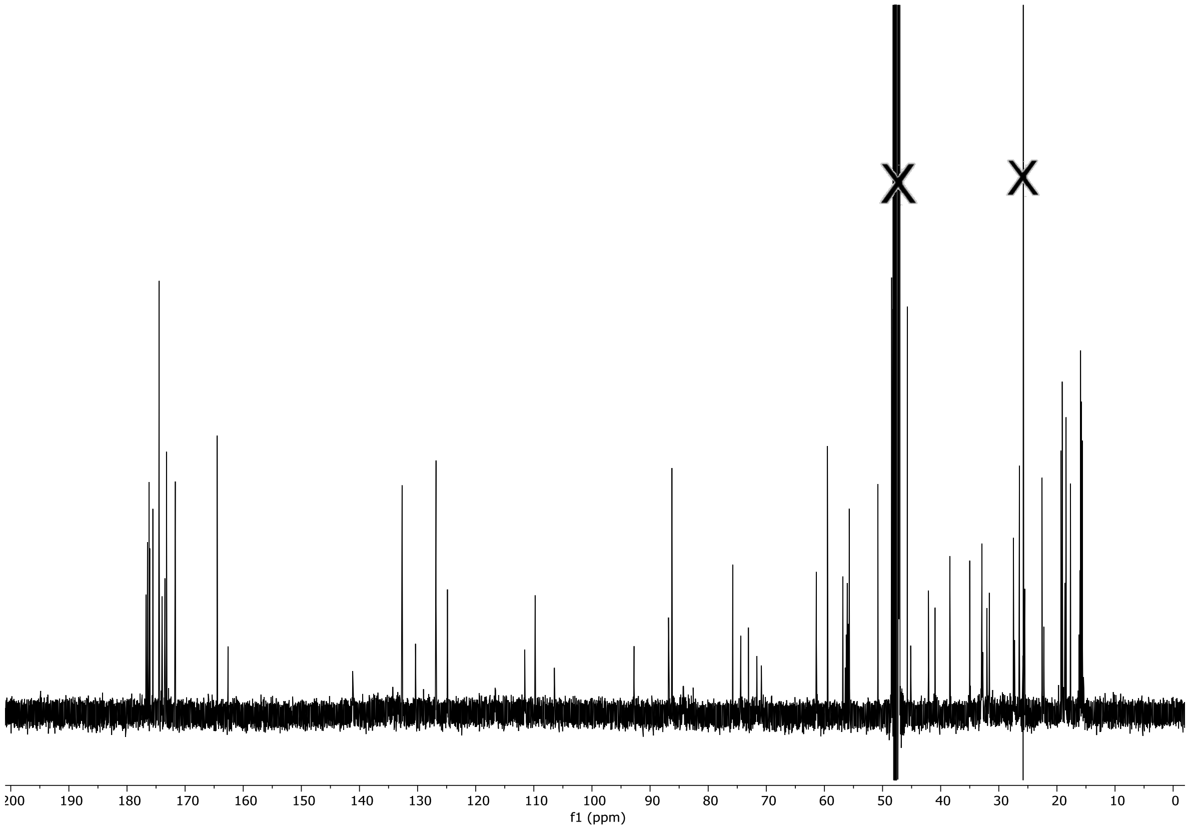

Figure S17. ^{13}C-NMR spectrum of **7** (126 MHz, CD_3OD (presat), 298 K, 8.7 x 10^{-3} M).

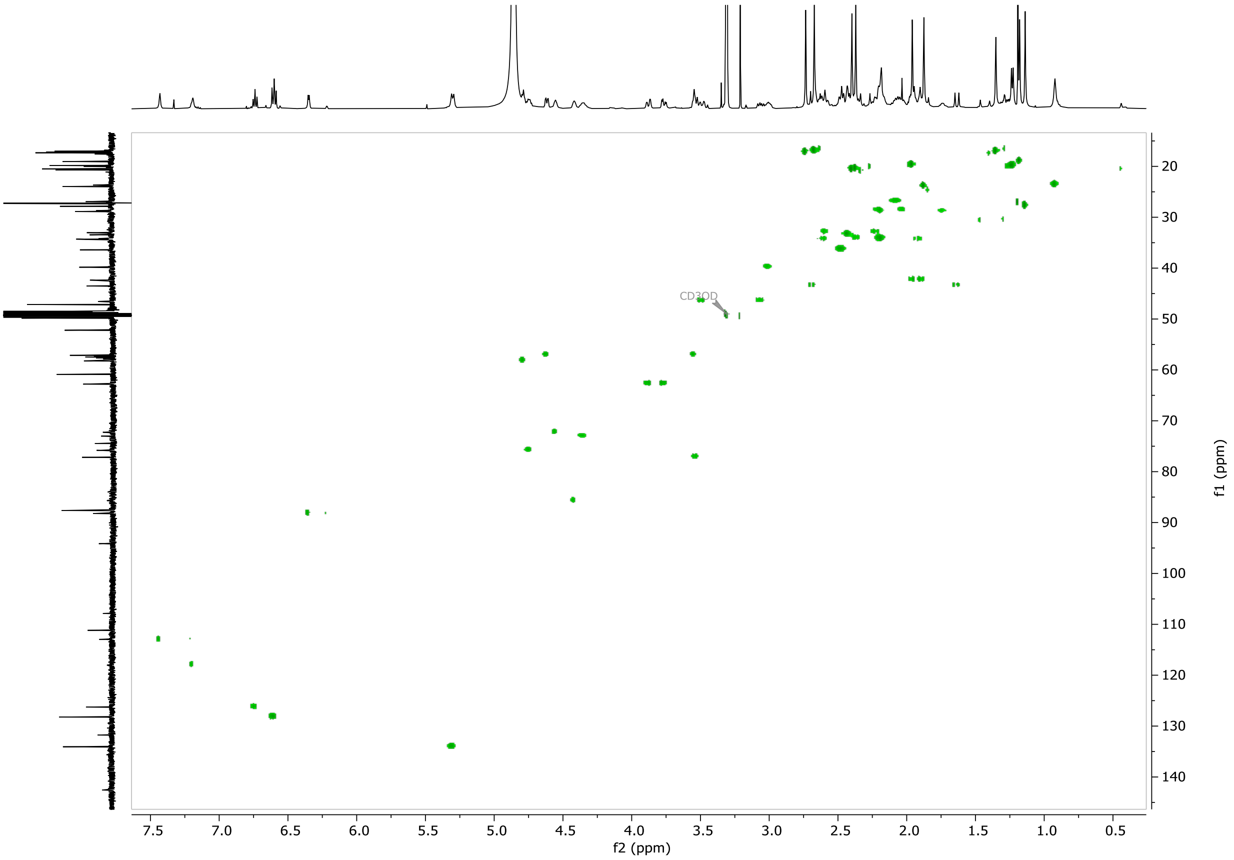

Figure S18. ^{1}H-^{13}C-HSQC spectrum of **7** (500 MHz, 126 MHz, CD$_3$OD , 298 K, 1024 x 1024 p, 32 scans, 8.7 x 10^{-3} M).

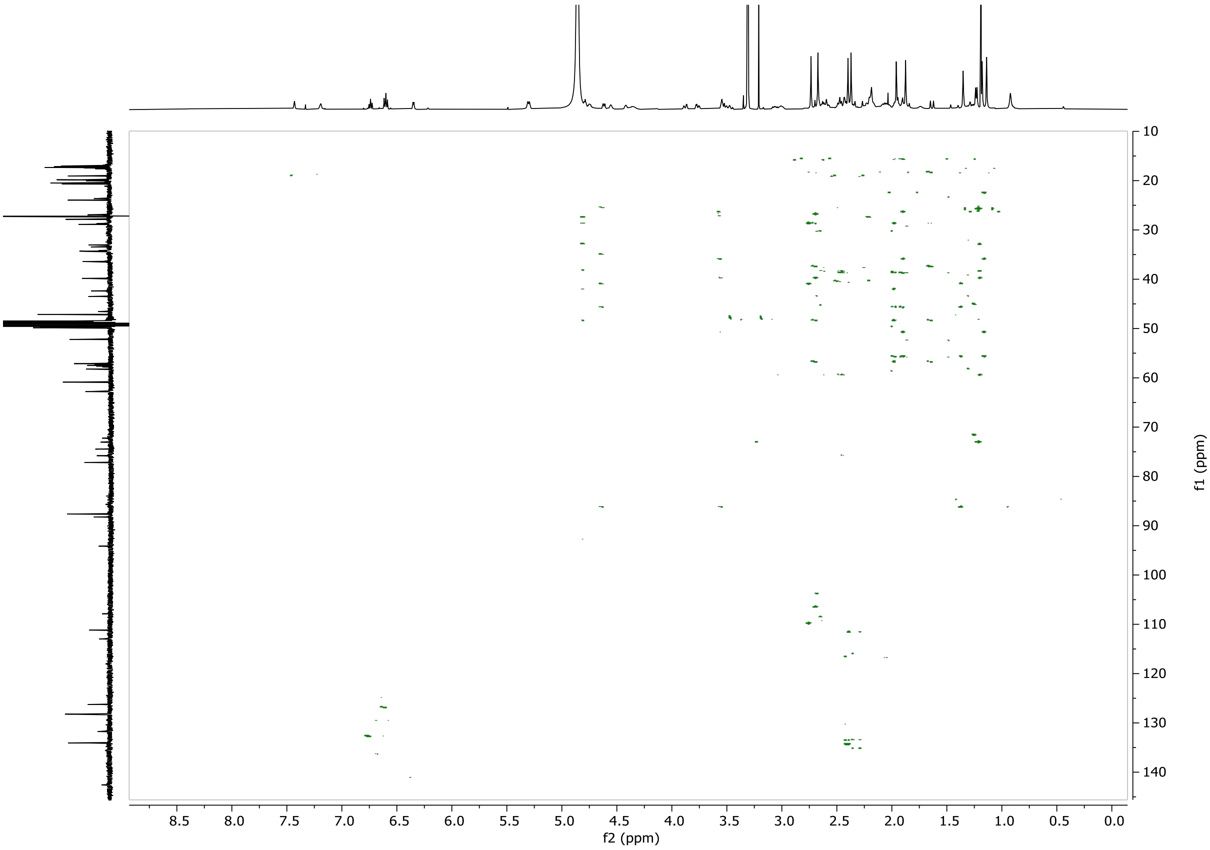

Figure S19. ^{1}H-^{13}C-HMBC spectrum of **7** (500 MHz, 126 MHz, CD$_3$OD, 298 K, 1024 x 2341 p, 12 scans, 8.7 x 10^{-3} M).

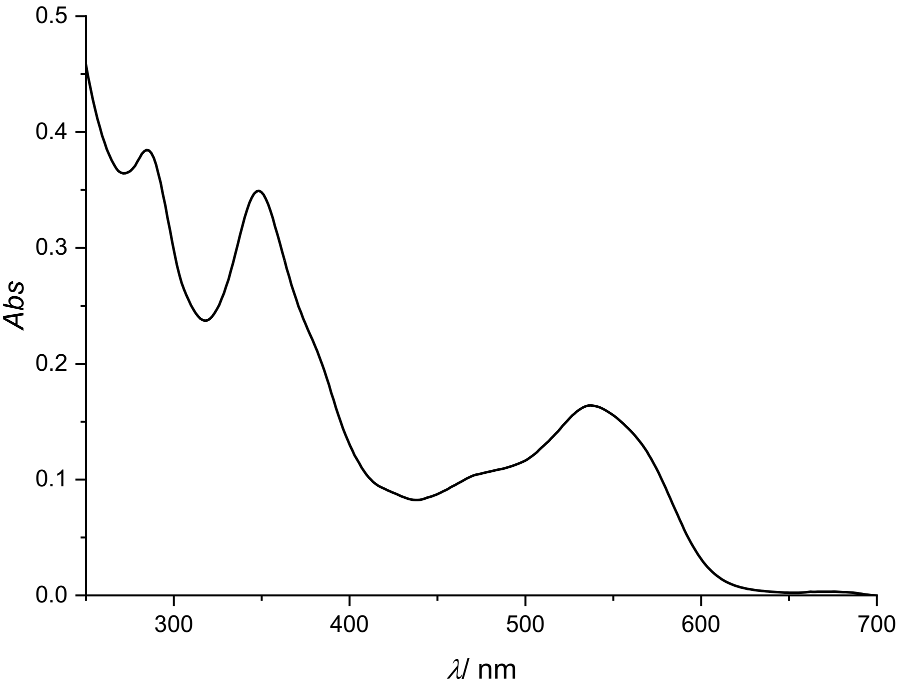

Figure S20. UV/Vis spectrum of **7** (H_2O, 3.5 x 10^{-5} M).

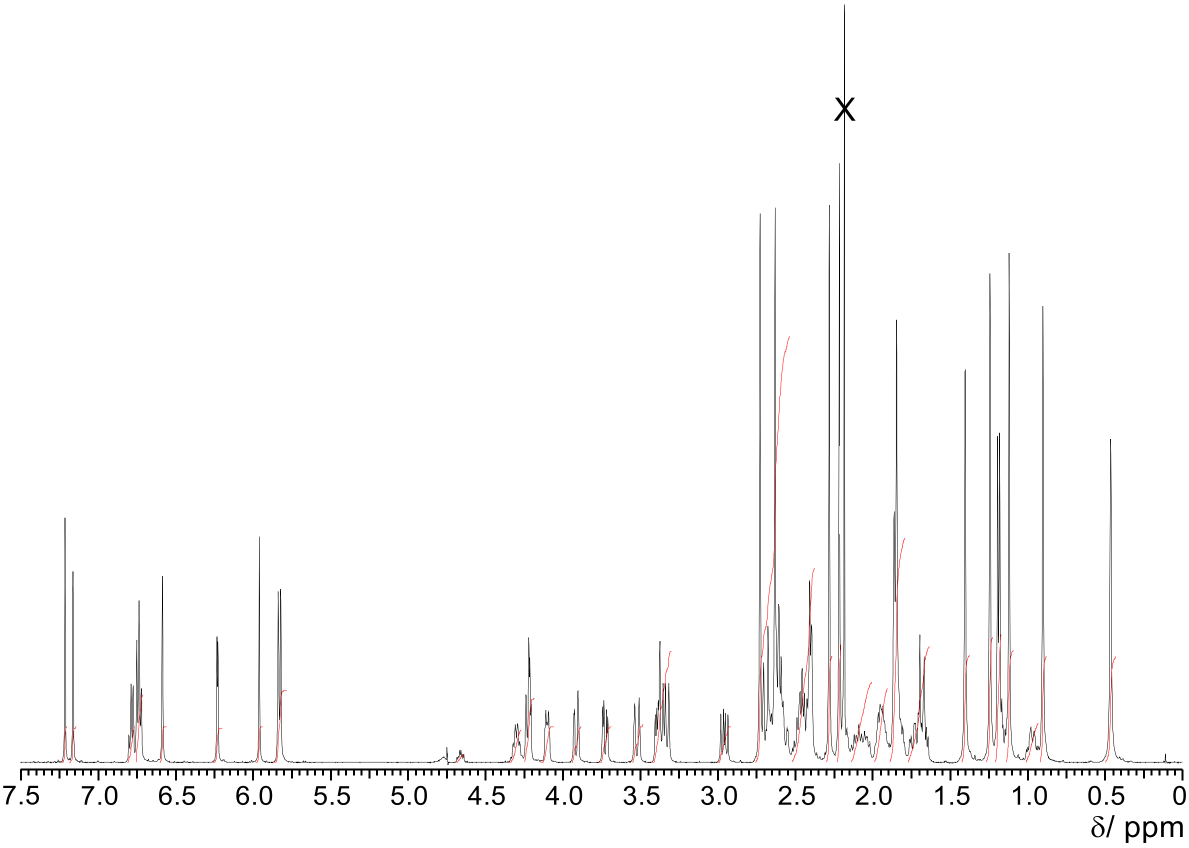

Figure S21. ^{1}H-NMR spectrum of **8**, **β-PhCbl** (500 MHz, D_2O (presat), 298 K, 3.2 x 10^{-3} M).

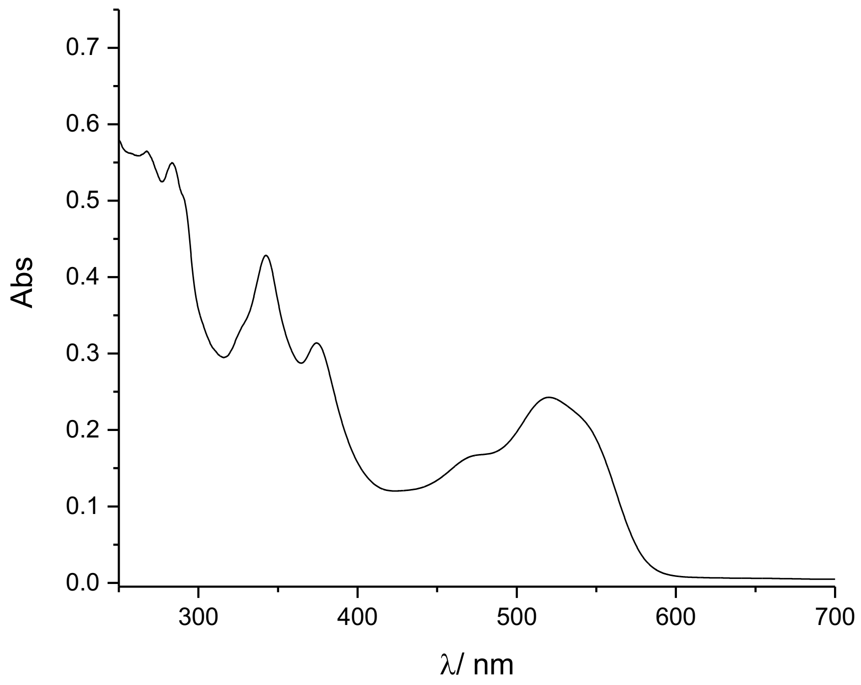

Figure S22. UV/Vis spectrum of **8, β-PhCbl** (H_2O, 6.3 x 10^{-5} M).
